# Corrosion potential and theoretical studies of fabricated Schiff base for carbidic austempered ductile iron in 1M H_2_SO_4_ solution

**DOI:** 10.1186/s13065-024-01278-0

**Published:** 2024-09-13

**Authors:** Ghalia A. Gaber, Lamiaa Z. Mohamed, Hayam A. Aly, Shimaa Hosny

**Affiliations:** 1 Department of Chemistry, Faculty of Science (Girls), Al-Azhar University, Yousef Abbas Str., P.O. Box: 11754, Nasr City, Cairo Egypt; 2 Mining, Petroleum, and Metallurgical Engineering Department, Faculty of Engineering, Cairo University, Giza, 12613 Egypt; 3 Central Metallurgical Research and Development Institute (CMRDI), Helwan, P.O. Box 87, Cairo, 11421 Egypt; 4 Department of Metallurgical and Materials Engineering, Faculty of Petroleum and Mining Engineering, Suez University, Suez, 43512 Egypt; 5 Chemistry Department, Faculty of Science, New Valley University, El-Kharga, 72511 Egypt

**Keywords:** Corrosion, Inhibitors, Carbidic austempered ductile Iron, Schiff base derivatives, Microstructure, Molecular modeling

## Abstract

In this body of work, a chemical known as 2-cyano-N-(4-morpholino benzyl dine) acetohydrazide (CMBAH) is explored for its ability to suppress the carbidic austempered ductile iron (CADI) corrosion in 1M H_2_SO_4_. Density functional theory was used in experiments and theoretical investigations to investigate the inhibiting impact. The corrosion of CADI alloys in 1M H_2_SO_4_ produced a corrosion resistance superior to that of CADI heat treatment (H.T.). As-cast carbidic ductile iron (CDI) 4 alloy with 1.5%t Cr-Nb has a corrosion rate (C.R.) of 11.69 mm/year, which drops to 5.31 mm/year at HT-275 °C and 6.13 mm/year at HT-375 °C. When describing the adsorption of inhibitors, the Langmuir adsorption isotherm is the most effective method. The findings of the G_ads_ show that the inhibition was induced mainly by the physisorption on the surface CADI alloys. In addition to this, it was found that the results of the experiments and the hypotheses were largely harmonious with one another. The formation of protective layers on the CADI surfaces is also visible in the images captured by the SEM. In 1M H_2_SO_4_, these Schiff base inhibitors effectively prevent corrosion caused by CADI. However, the combination of inhibitors leads to a fine microstructure with ausferrite and narrow ferrite needles, promoting corrosion resistance. The CADI needles rated an upper ausferritic microstructure with wide ferrite needles.

**Supplementary Information:**

The online version contains supplementary material available at 10.1186/s13065-024-01278-0.

## Introduction

Ductile iron (DI) is a low-cost engineering material with superior characteristics because of these features, it is appropriate for a vast array of uses, comprising tubes, machine parts, and automotive, as well as in conditions requiring high corrosion resistance against soils, water, alkalis, acids, saline solutions, organic chemicals, and liquid metals [1]. Random distribution of graphite spheres in a dual-phase matrix [ferrite (α-phase) and high carbon austenite (noted γHC-phase)] in austempered ductile iron (ADI). Alloying chemicals may be the most important aspect of ADI's vulnerability to corrosion assaults. Si, Ni, Cr, and Cu are common alloying elements that enhance ADI corrosion resistance. Additional alloying elements employed to a lesser degree comprise V, Ti, and Mo [2]. Austempering of DI has been extensively investigated. However, the same technique was used for Cr and Nb. Carbidic austempered ductile iron (CADI) is a specialized type of ADI characterized by its relatively high carbon content [3]. CADI has a superior combination of hardness and toughness than ADI due to improved carbide morphology and carbide type shift [4]. Cr-bearing DI features tough and wear-resistant M_7_C_3_ carbides and is commonly used in various hard-facing applications.

The resultant ADI has double the strength of traditional DI for the same elongation level and outstanding wear and fatigue resistance [5]. The fundamental issue observed throughout eutectic solidification is the expansion of M_7_C_3_ carbide extraordinarily quickly along its favored growth axis, resulting in laths or rod morphology. Controlling the basic carbide shape thus entails changing the development and increasing the nucleation by novel alloying procedures (possibly by adding an element that inhibits the growth of M_7_C_3_ carbide but not its nucleation). Controlling eutectic colony size improves CADI’s mechanical and wear properties [6]. Carbide structural adjustments increased toughness, whereas abrasion resistance was increased using even harder carbides. The alloy’s microstructure influences corrosion behavior, like mechanical qualities. A literature review shows substantial consideration has been given to DI and ADI corrosion resistance, mostly alloyed with Ni [7]. In addition, Ni reduces primary carbide stability while increasing pearlite's finesses, enhancing the iron’s strength [8]. The ASTM A439 standard specifies high-Ni DI for its heat, corrosion resistance, and other applications [7]. Investigations on cast irons (CI.s) and austempered chilled DI corrosion, including Ni, show that corrosion decreases as the Ni level increases [9].

The corrosion characteristics of each cast iron (CI) class vary widely. Still, single phases like ferrite and austenite are generally less corrosion-resistant than mixtures of the two-phase (e.g., pearlite and ausferrite) [10]. ADI austempered at temperatures between 250 and 330 °C provides a lesser ausferritic microstructure, while high austempered temperatures (330 °C–450 °C) yield higher ausferritic microstructures with wide blades of isolated ferrite; this microstructure produces in an excellent tensile toughness [11]. Electrochemical techniques assessed ADI’s electrochemical behavior and corrosion resistance in NaCl. The corrosion resistance of ADI with an upper ausferritic microstructure is stronger than that of ADI with a lower ausferritic morphology [3]. Mo was discovered to significantly improve the corrosion resistance of DI, particularly in high-Si DI alloys [12]. Furthermore, the mix of Ni and Mo levels in austempered chilled DI and chilling austempering contribute to its exceptional corrosion resistance [12]. Chronopotentiometry and potentiodynamic techniques were employed to assess the corrosion behavior of DI and ADI at low NaCl concentrations. Both samples were found to be subjected to similar corrosion deterioration; however, the austempering heat treatment (H.T.) aids in the stabilization of nodules, enhancing their corrosion resistance in corrosive environments. Sulphuric acid (H_2_SO_4_) is frequently utilized in many industrial operations. There are only a select few alloys that are acceptable for usage in H_2_SO_4_ media, such as cast iron or stainless steel. Steel parts are washed with, or rinsed in acidic solutions, mainly sulfuric acid, during maintenance activities to clean the surface and remove rusts and calcifications. The drawback of this important procedure is the violent acidic attack's corrosion of the metal surface, which results in flaws or even permanent damage to the machine parts. To overcome this issue, chemical compounds known as corrosion inhibitors are added to acidic solutions to reduce the rate of corrosion to a minimum [13].

Schiff bases, an important class of chemical compounds, prevent acidic corrosion of CADI. Thus, adding some organic corrosion inhibitors to the acidic solution can eliminate austempered ductile iron corrosion. Adding an inhibitor to the corrosive solution has created vital metal corrosion protection technologies. It is a simple application, low cost, essential technology, and significant protection. N, O, S, P, sigma bonds, functional groups, and heteroatoms characterize organic inhibitors [14]. The organic compound’s corrosion inhibitors’ lone pair electrons can form a dative covalent connection with the transition metal’s vacant orbital, overcoming strict chemisorption [15]. These heteroatoms and their conjugated structure adsorb organic molecules (inhibitors) to metal surfaces. This adsorption might be physical (physisorption) through electrostatic contact between the metal surface and inhibitor molecules or chemical (chemisorption) by the establishment of a coordinate bond between the metal’s vacant d-orbital and inhibitor [16, 17]. Active functional groups like hydroxide, cyanide, imine, or carbonyl, aromaticity, and electron density can affect metal-inhibitor molecule interactions [18]. Many synthetic chemicals inhibit acidic environments, including quinolone [19], hydroxyquinoline [20], thiourea [21], urotropine [22], imidazole [23], thiadiazol [24], dihydropyrazole [25], and Schiff base (S.B.) [26]. Adsorption of chemical inhibitors at the metallic contact forms a protective layer. Schiff bases made from amino acids include phenolic, –C=N, and carboxylic hydroxides, making them promising corrosion inhibitors [27].

Schiff bases are more prevalent as anticorrosion inhibitors than other chemical compounds [28]. A Schiff base derived from 4-morpholinobenzaldehyde is a new inhibitor for the corrosion of CADI in 1M H_2_SO_4_ Solution. Several publications have explained the corrosion characteristics of ADI in bulk electrolyte solutions [29, 30]. Fewer research publications on the inhibitory effect on corrosion performance in CDI and CADI. The current study seeks to investigate corrosion inhibition of CADI by 2-cyano-*n*-(4-morpholino benzyl dine) acetohydrazide (CMBAH) in 1M H_2_SO_4_ corrosive media utilizing weight loss (W.L.) estimation. Corroded CDI and CADI's surface morphology was examined by scanning electron microscopy (SEM).

## Experimental work

### Synthesis of Schiff base inhibitor (SBI)

**Fig. 1:**
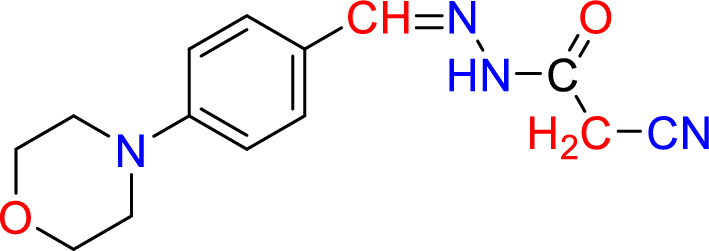
Structure of SBI

The SBI was prepared, and its purity was established according to previously described methods [31]. The molecular structure of Schiff bases is presented in Fig. 1.

### Preparation of the working electrode

#### CDI fabrication and H.T.

The chemical composition of the specimens is examined using a vacuum Oxford Pro spectrometer, shown in Table 1. The phase identification of the CDI 4 alloy was performed by X-ray diffraction (XRD) model X’PERT PRO. PANLYTICAL device.

**Table 1:** Composition of the investigated CDI alloys

Alloy No		Chemical compositions, %								
		C	Si	Mn	S	P	Mg	Cr	Nb	Fe
CDI 1	1% Cr	3.50	2.51	0.22	0.03	0.03	0.05	1.04	0	Bal
CDI 2	1.5% Cr	3.55	2.61	0.22	0.02	0.02	0.05	1.05	0	Bal
CDI 3	1% Cr–1%Nb	3.59	2.53	0.22	0.02	0.03	0.05	1.00	1.0	Bal
CDI 4	1.5% Cr–1%Nb	3.60	2.50	0.20	0.02	0.02	0.05	1.52	1.1	Bal

The H.T. of the four samples creates austempered CDI that enhances the specimens’ mechanical characteristics with little deformation and stresses. Austempering H.T. is a two-step procedure. During the first step, the specimens are heated to the austenitizing temperature (900 °C) and kept for 1 h. In the second step, specimens are swiftly cooled from austenitizing to austempering temperatures (275 °C and 375 °C) and then immersed for 1.5 h. CDI 1 develops CADI1, CDI2 becomes CADI2, CDI3 converts CADI3, and CDI4 becomes CADI4 after austermpering. The SEM for the CDI alloys were examined.

### Weight loss measurements

The CDI and CADI alloys were utilized for W.L. evaluations, which were conducted following prior reports of one of the authors [32]. A rectangular CADI sample 20 × 20 × 5 mm was stood with 600, 800, and 1000 grit grinding papers, cleaned with double distilled water, degreased with acetone, dried at R.T., and weighed. After weighing accurately, the specimens were submerged in 100 mL beakers, including 1M H_2_SO_4_ solution with inhibitor concentrations (50 ppm and 100 ppm) for 5 days. Corrosion inhibitors were 2-cyano-*N*-(4-morpholino benzyl dine) acetohydrazide. The trials were conducted three times, and the average data were used in the computations. The change in the weight is calculated from Eq. 1, and the C.R. is calculated from Eq. 2 [33].

1 $$\Delta {\text{W }} = {\text{ W1 }} - {\text{ W2}}$$

2 $${\text{C}}{\text{.R}}{.}\left( {\text{mm/y}} \right) = \frac{{\Delta {\text{W}} \times {\text{ K}}}}{{{\text{A}} \times {\text{T}} \times {\text{D}}}}$$

K denotes 8.76 × 10^4^, T represents the time of exposure, A denotes area in cm^2^, ∆W indicates weight reduction in g, and D describes density in g/cm^3^ for alloys.

The efficiency (I.E.) and surface coverage (θ) are found in Eqs. 3 and 4:

3 $$\% {\text{IE}} = \left( {{\text{CR}}_{0} - {\text{CR}}_{{{\text{inh}}}} /{\text{CR}}_{0} } \right) \times {1}00$$

CR_0_ and CR_inh_ are the C.R. values of CADI with/without SBIs, respectively [34].

4 $$\uptheta = {\text{IE}}/{1}00$$

### Potentiodynamic polarization

Potentiodynamic polarization (PDP) curves were recorded by scanning the electrode potential from − 0.8 to + 0.5 V at 5 mVs^−1^. Corrosion inhibition was measured using a laptop and a VoltaMaster PGZ 301 potentiostat/galvanostat with a three-electrode cell assembly. This procedure’s cell has the working electrodes (CDI), counter PT, and reference SCE. Tafel polarization analysis of anodic and cathodic curves calculated current densities (I_corr_). The C.R. is calculated from Eq. 5 [35]

5 $${\text{CR}}\left( {\upmu {\text{m}}/{\text{y}}} \right) = {3}.{3}Icorr{\text{M}}/{\text{zd}}$$

where z is ionic charge, M represents atomic weight of metal, d is density g/cm^3^, and *I*_*corr*_ represents corrosion current density, μA/cm^2^.

### Computational details

SBI was optimized with the Gauss View 09 program [36], based on B3LYP, which is functional with 6-311++G basis set. Default convergence criteria was applied. Frequency analysis was used to test the geometry optimizations. Calculations of frequency showed that imaginary frequency was absent.

### Surface analysis

The S.M. of the corroded CADI alloys in 1M H_2_SO_4_ with/without 100 ppm of inhibitors was characterized utilizing JOEL 7600 F.

## Results and discussion

### Phase identification

Figure. S1 shows the XRD pattern of the CDI 4 alloy. There are three main phases: α-iron, chromium (M_7_C_3_) and niobium carbides.

### Microstructure before corrosion

**Fig. 2:**
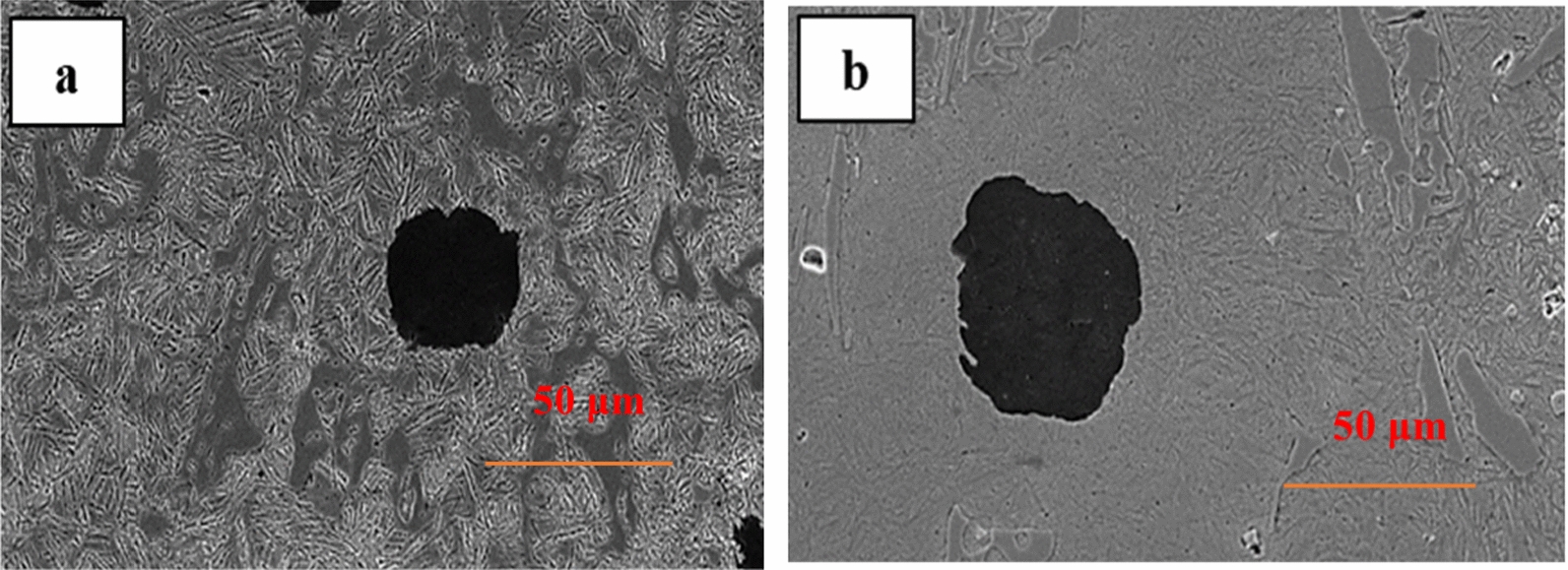
SEM images of CADI 4 alloys at different H.T. **a** 275 °C, and **b** 375 °C

Figure 2a and b show the SEM images of CADI 4 alloys at 275 °C and 375 °C, respectively. The SEM images of CADI 4 alloys of various H.T.s consist of graphite spheroids and carbides in the ausferrite matrix.

### Weight loss measurements

#### The behavior of CADI alloys in 1M H_2_SO_4_ at 25 °C

**Fig. 3:**
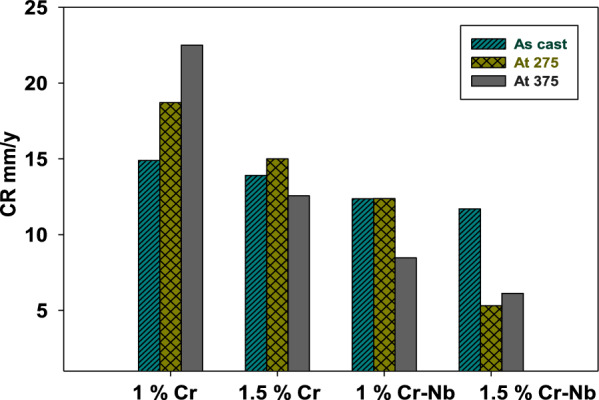
Comparing the CR of alloys in 1M H_2_SO_4_ after 5 days immersion time

The W.L. test of CDI and CADI alloys obtained in 1M H_2_SO_4_ solutions are shown in Fig. S2. The values of W.L. (g) of CADI samples in 1M H_2_SO_4_ at different immersion times for as cast, H.T.-275 °C and HT-375 °C are presented in Fig. S2. Inspection of the presence of Cr and Nb caused a considerable drop-in dissolution rate and a comparable corrosion rate (C.R.) decrease. The reduction in C.R. may be ascribed to the alloy’s contact with the iron via adsorption, which blocks more active corrosion sites [37]. The effect of immersion time on CADI alloys in 1M H_2_SO_4_ was studied. W.L. increased for 5 days of submerged time. This is owing to an adsorption–desorption process. The samples were submitted to mass loss studies at extended immersion durations to detect the protective trend. The addition of chromium to CADI (with 1% and 1.5% Cr) improves corrosion resistance by fostering the formation of a protective chromium oxide layer on the surface. This layer serves as a barrier, minimizing the metal’s exposure to the acidic environment and reducing corrosive damage. Similarly, the inclusion of niobium (with 1%-Nb and 1.5% Cr-Nb) enhances corrosion resistance by creating a more stable and protective oxide layer, which shields the metal from direct contact with sulfuric acid and lowers the corrosion rate. Increasing both chromium and niobium in Carbidic Austempered Ductile Iron (CADI) markedly affects its corrosion performance in sulfuric acid. The W.L. (g) and C.R. (mm/y) values are presented in Table S1, while Fig. 3. represents the comparing C.R. of CADI samples in 1M H_2_SO_4_ after 5 days of immersion time. In addition, the C.R. of CADI 1 (1% Cr) increases from nearly 14.90 mm/y up to 22.50 mm/y for as cast and HT-375 °C, respectively. This tendency indicates that surface activity relates to developing a porous rust layer at the s CADI surface. Frequently, pores and fissures are apparent in this stratum. On iron, it has previously been proven that porous rust may operate as a water retention phase [38] and absorb Cl^−^ions [39], which can speed up the C.R. [40]. The rust layer of high porosity (outward oxidation) progressively covers all graphite spheres. The C.R. in the case of CADI 4 (1.5% Cr-Nb) is 11.69 mm/y for as-cast alloy and decreased to 5.31 mm/y for HT-275 °C and 6.1264 mm/y at HT-375 °C. Iron corrosion in an acidic medium [41], increases the reaction rate when the pH drops, which is proportional to the rate of hydrogen evolution that determines C.R. M H_2_SO_4_ This occurs for CADI 1 As cast and heats (1% Cr), where the corrosion current increases in 1. Suppose one considers that graphitic corrosion is generated mainly by the potential difference between graphite and the surrounding ferrite. In that case, the graphite-ferrite potential difference is negligible [42], then iron dissolution results from reactions (6) and (7):

6 $${\text{Fe}} \to {\text{Fe}}^{{{2} + }} + {\text{2e}}^{ - } \quad \quad {\text{Anodic}}$$

7 $${\text{2 H}}^{ + } + {\text{2e}}^{ - } \to {\text{H}}_{{2}} \quad \quad {\text{Cathodic}}$$

In terms of lowering corrosion resistance, H_2_SO_4_ acid is the most aggressive. This is because when pH falls, the equilibrium potential of reaction 6 gets more noble; thus, the potential difference between reactions 1 and 2 becomes greater, and the corrosion current rises [42]. In acid media (H_2_SO_4_), two dissolving processes may occur; the first is due to the sulfur ion, while the second is due to oxygen [43]. Both enhance the rate of disintegration of CADI. Reactions from 8 to 10 constitute the dissolving process in acidic environments [41].

8 $${\text{2Fe}} + {\text{3H}}_{{2}} {\text{SO}}_{{4}} \to {\text{Fe}}_{{2}} {\text{O}}_{{3}} + {\text{3SO}}_{{2}} + {\text{3H}}_{{2}} {\text{O}}$$

9 $${\text{2Fe}} + {\text{3H}}_{{2}} {\text{SO}}_{{4}} \to {\text{2FeOOH}} + {\text{3SO}}_{{2}} + {\text{2H}}_{{2}} {\text{O}}$$

10 $${\text{Fe}} + {\text{H}}_{{2}} {\text{SO}}_{{4}} \to {\text{FeO}} + {\text{SO}}_{{2}} + {\text{H}}_{{2}} {\text{O}}$$

As the reaction mechanisms progress, a hydroxide layer is created, the passage of oxygen across which creates corrosion resistance. The dissolution of this layer in acidic environments increases the C.R.[41].

#### Inhibition action of CADI alloys in 1M H_2_SO_4_ + 50 ppm at 25 °C

The CMBAH was tested against corrosion of CADI alloys in 1M H_2_SO_4_. The effect of two concentrations of inhibitors (50 and 100 ppm) was studied in 1M H_2_SO_4_. Firstly, the impact of 50 ppm of inhibitors on CADI alloys using W.L. tests as shown in Fig. S3 are tabulated in Tables S2, S3, and S4 for as cast, HT-275°C, and HT-375 °C, respectively.

The CMBAH displayed a rise in the I.E. % upon raising the inhibitor concentration irrespective of the corrosion environment, as described in Table S5. This occurs because of enhanced surface coverage of inhibitor molecules. The C.R. data of the examined inhibitors in 1M H_2_SO_4_ corrosion media with 50 ppm for CAI4 and CADI 4 (1.5% Cr-Nb) are 10.2166 mm/y as cast, 4.983 mm/y at HT-275 °C, and 3.67 mm/y at HT-375 °C. The SBIs are the best value against corrosion in an acidic medium, as provided in Table S5.

It is observed that the lowest C.R. for all alloys as cast or H.T. is in the presence of inhibitors evaluated compared to CADI alloys without inhibitors. The CADI sample has coarse graphite nodules. However, adding Cr and Nb or inhibitors results in a refined microstructure and enhancement in the number of nodules encircled by ferrite and fine pearlite. This decreases corrosion resistance because the galvanic graphite-ferrite combination facilitates corrosion, also known as graphitic corrosion [42]. However, it should be noted that the CADI sample has a small number of graphite nodules enveloped by ferrite, and the microstructure etched with (NH_4_)_2_S_2_O_8_ demonstrates that CADI is rich in carbides. The greater the concentration of carbides, the greater the corrosion resistance [44, 45]. However, certain applications would not benefit from the presence of carbide since it causes fragility.

CADI 4 HT-275 °C and HT-375 °C have a lower CR than CADI 3 HT-275 °C and HT-375 °C, indicating that they possess stronger corrosion resistance. According to microstructural analysis, both contain graphite surrounded by ferrite, which produces the galvanic pair that promotes graphite corrosion. Still, while corrosion occurs on the metal's contact surface, the benefit of CADI 4 (1.5%Cr-Nb) is that its tiny microstructure, which creates fewer graphite nodules, also decreases the surrounding ferrite. Additionally, pearlite increases, diminishing the corrosion current in acidic conditions.

#### Inhibition action of CADI alloys in 1M H_2_SO_4_ + 100 ppm at 25 °C

**Fig. 4:**
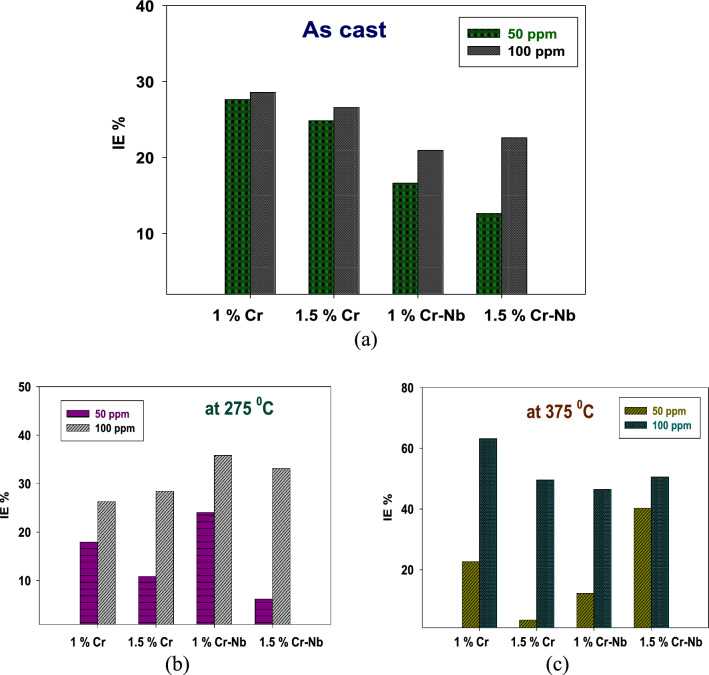
Variation of I.E. % for alloys in 1M H_2_SO_4_ with of CMBAH **a** As cast, **b** HT-275°C, and **c** HT-375°C

The effect of 100 ppm of inhibitors on CADI alloys using W.L. tests are tabulated in Tables S6, S7, and S8 for as cast, HT-275 °C, and HT-375 °C, respectively. The values of W.L. (g), C.R. (mm/y), and inhibition efficiency are provided in Table S9, whereas Fig. S3 illustrates the change of W.L. versus time rates and inhibition efficiencies (percent I.E.) for all CADI alloys. W.L. measurements determined that the dissolution rate decreased significantly across a concentration range, accompanied by a rise in the inhibitors' efficiency (I.E. percent), as seen in Fig. 4. The reduction in C.R. and increase in efficiency percent with the application of inhibitors may be related to the contact of inhibitor molecules with the steel surface through an adsorption process, which blocks more active corrosion sites [37]. At a concentration of 100 ppm, the highest inhibitory effect was seen.

Figure. S4 shows the CR of the CADI evaluated in 1M H_2_SO_4_ corrosive media with 100 ppm of investigated inhibitors. The findings generally indicate an improvement in corrosion resistance relative to CADI 4. Where the corrosion current is increased, however, the corrosion potential tends to move to positive values, suggesting a decreased susceptibility to rust. This is related to a higher quantity of acicular ferrite embedded in an austenite matrix, and these phases prevent graphitic corrosion.

CADI 4 exhibits greater corrosion resistance than CADI 1 because of the phase transition induced by H.T., which shifts the corrosion potential to more positive or noble values. The CADI produced contains both ausferrite and high-carbon austenite. Carbon dissolution in austenite reduces the impact of a potential difference between graphite and austenite, reducing the C.R. CADI 4, which has the greatest corrosion resistance in acidic conditions. This behavior results from the ausferrite’s tiny microstructure generated by combining Cr, Nb, and inhibitors.

### PDP measurements

#### Corrosion of CADI alloys in 1M H_2_SO_4_ at 25 °C

**Fig. 5:**
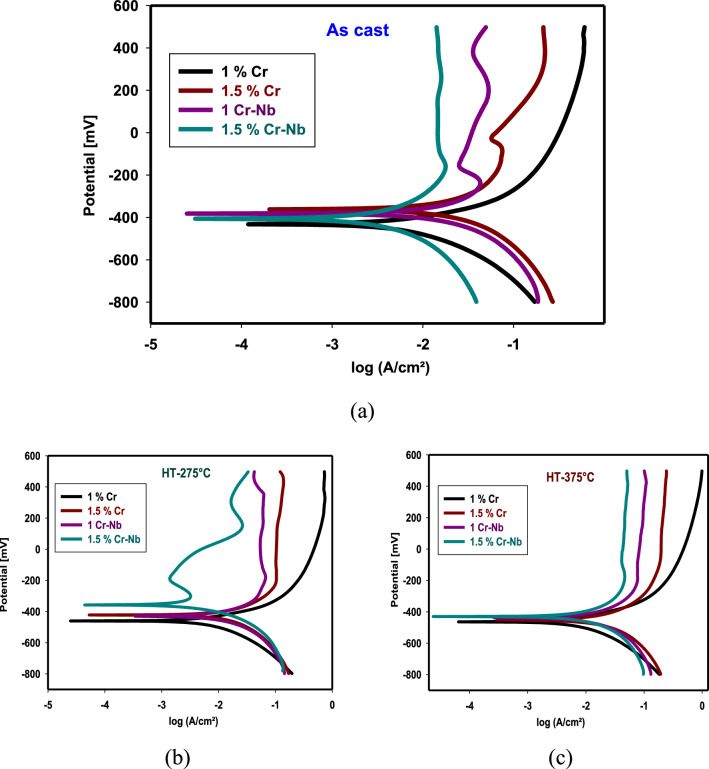
Potentiodynamic polarization curves for alloys in 1M H_2_SO_4_ **a** As cast, **b** HT-275°C, and **c** HT-375 °C

The polarization plots of CDI and CADI alloys in 1M H_2_SO_4_ solutions are given in Fig. 5. Tables S10, S11, and S12 show potential corrosion (E_corr_), Tafel slopes (βa, βc), current density (I_corr_), and CR of CADI samples in 1M H_2_SO_4_ for as cast, HT-275 °C, and HT-375 °C. Data show that adding Cr to CADI alloys lowers I_corr_, and adding Cr and Nb maintains this decrease. Adsorption of iron by the alloy may reduce C.R. by blocking more active corrosion sites. For as cast and HT-375 °C, the C.R. of CADI 1 (1% Cr) rises from about 204.7 mm/y to 193.8 mm/y. Surface action seems to generate a porous rust coating on the CADI surface. CADI 4 (1.5% Cr-Nb) has a C.R. of 61.68 mm/y for as-cast alloy, 38.92 for HT-275 °C, and 31.85 for HT-375 °C. The changes in the slopes of the anodic and cathodic Tafel lines (βa and βc) indicate that the inhibitors affect the dissolution mechanism of CDAI. The behavior of CDAI in the presence of inhibitors displayed an active–passive behavior. This is evident from the cathodic current–potential curves, which show parallel Tafel lines and a decrease in cathodic current density with inhibitors, while the anodic portions are only slightly affected. This suggests that the inhibitor adsorbs onto the metal surface at the cathodic sites, leading to inhibition, while it also reduces the anodic current density [46].

#### Corrosion inhibition of CADI alloys in 1M H_2_SO_*4*_ with CMBAH

**Fig. 6:**
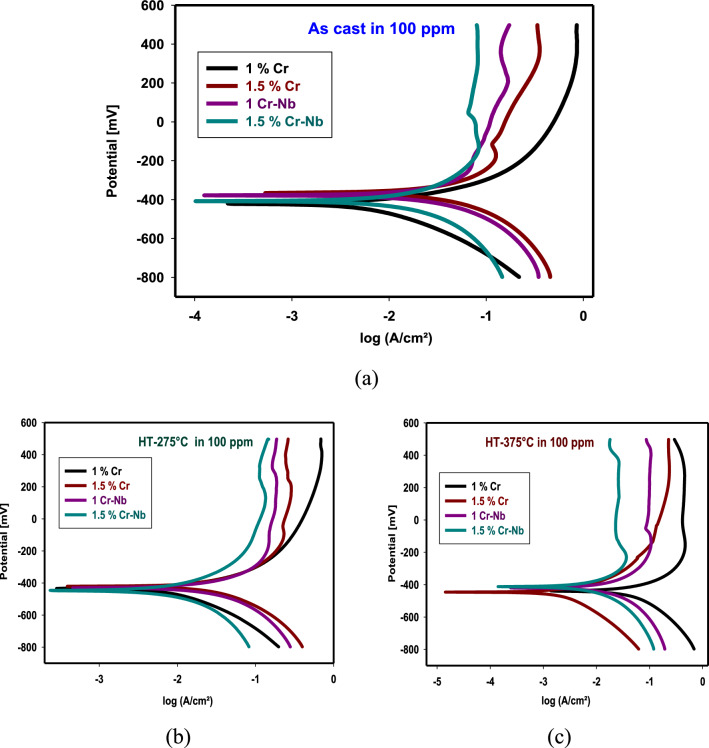
The PDP curves for alloys in 1M H_2_SO_4_ with 100 ppm of investigated inhibitor **a** As cast, **b** HT-275 °C, and **c** HT-375 °C

Figure 6 shows polarization curves of inhibitors on CADI alloys at as cast, HT-275 °C, and HT-375 °C. Tables S13, S14, and S15 show potential corrosion (E_corr_), Tafel slopes (βa, βc), current density (I_corr_), and CR for as cast, HT-275 °C, and HT-375 °C. The results show that inhibitors lower I_corr_ and C.R. I_corr_ decreases because inhibitor chemicals adsorb on CADI alloys and block surface active sites. Table S16 displays the surface coverage (θ) and efficiency (I.E. percent). Also, these studies illustrate how well the inhibitors cooperate to halt CADI alloy corrosion. Therefore, anodic, and cathodic reactions are greatly impeded. Inhibitors affect cathodic and anodic processes, but mostly anodic reactions. Due to the phase shift generated by H.T., CADI 4 has greater corrosion resistance than CADI 1. The generated CADI contains ausferrite and high-carbon austenite. Carbon dissolving in austenite lowers the C.R., reducing the influence of graphite-austenite differences. CADI 4 resists acid corrosion best. The nanoscale microstructure of ausferrite, formed by fusing Cr, Nb, and inhibitors, produces this behavior.

Since cast iron contains more carbon than low-carbon steel, it is corroded more quickly in diluted acid. The hydrogen evolution reaction is supported by carbides (noble), whereas the ferritic phase (matrix) anodically dissolves in carbon steels [47]. If a high potential is supplied, the surplus water molecules in diluted sulfuric acid may be oxidized, leading to the formation of a non-adherent oxy-hydroxide coating. It may be possible to make advancements in the design of corrosion-resistant materials that are specifically tailored for sulphuric acid environments by conducting more studies in this area. By creating more cost- and environment-friendly, long-lasting, and reliable corrosion protection techniques. For instance, the creation of fresh corrosion inhibitors that can better safeguard metal alloys in sulphuric acid medium while also being safe to handle and sustainable should be prioritized. The effectiveness of Gemini surfactant (GS), as a corrosion inhibitor for mild steel in 1 M sulphuric acid was tested in the study carried out by Haladu et al. [48].

### Adsorption isotherm (A.I.) studies and thermodynamic parameters

The A.I.s are essential for establishing corrosion inhibition because they reveal the molecular interaction between the inhibitor and active spots on the cast iron surface. The degree coverage (**θ**) was estimated from the mass loss measurements for CADI alloys. Various A.I.s, containing Langmuir, Temkin, and Freundlich, were used to examine data visually. LAI (correlation coefficient R^2^ equal to 0.9930) gave the predicted linear connection through surface coverage of adsorbed inhibitor on the alloy surface.

### Langmuir adsorption isotherm (LAI)

**Fig. 7:**
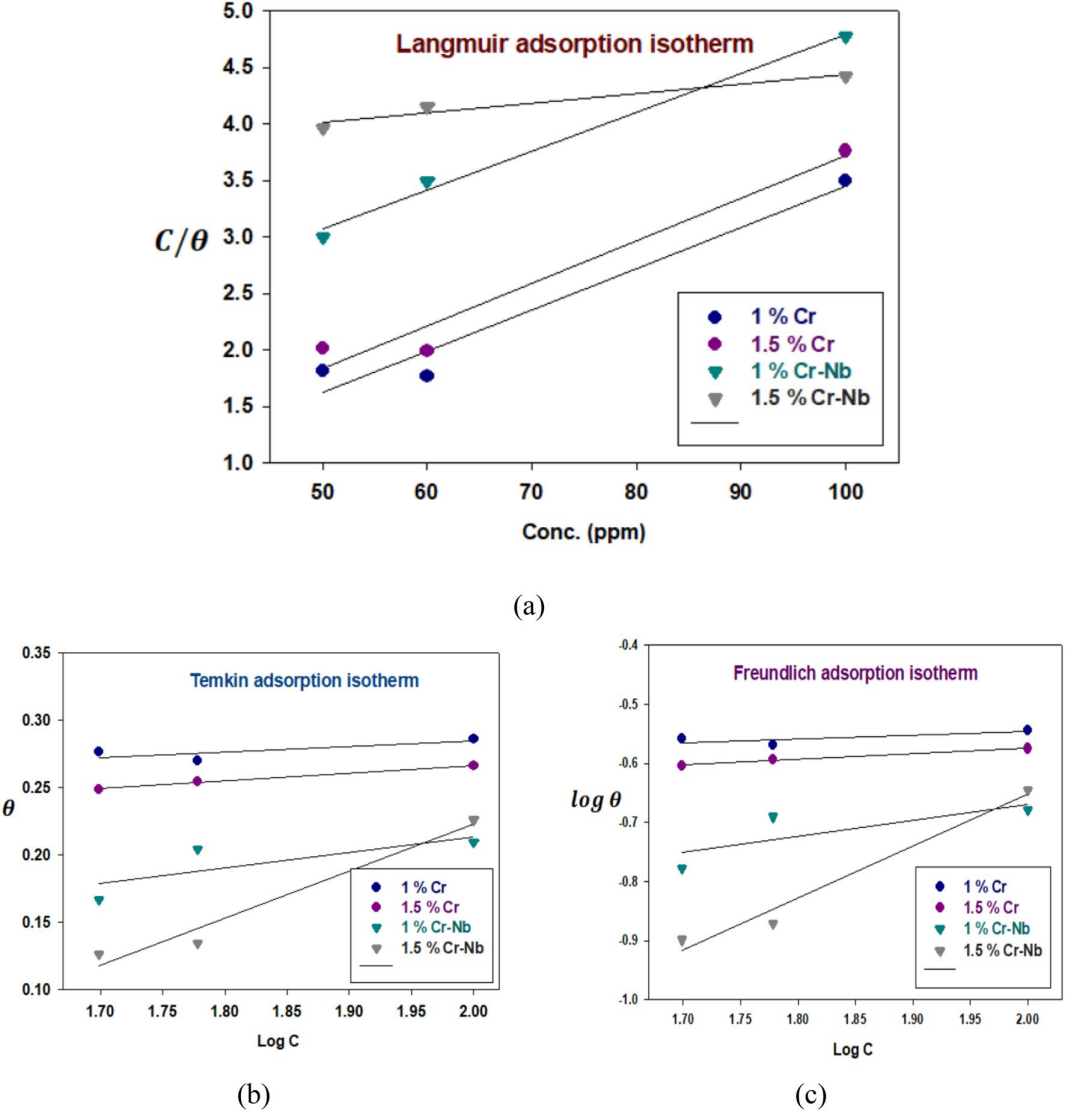
A.I. plots from W.L. measurements for the corrosion of CDI alloys as cast in 1M H_2_SO_4_ solution in the absence/presence of concentrations of inhibitor at 25 °C

The values of surface coverage were graphically determined from the W.L. method through a suitable fitting of A.I. to scrutinize the adsorption process of the inhibitors on CADI alloys. The best relation was attained from the LRI [49, 50] through Eq. 11:

11 $$\frac{{\text{C}}}{{\uptheta }} = {1}/ {\text{K}}_{{{\text{ads}}}} + {\text{ C}}$$

where C denotes the inhibitor concentration and ${\text{K}}_{\text{ads}}$ is defined as a constant of the inhibitor adsorption. Figure 7a displays a plot between C/$\uptheta$ as the X-axis against C as the Y-axis, resembling the LAI. A perfect linear plot was produced with a regression constant ${\text{R}}^{2}$ = 0.9930. It is customary that ∆${\text{G}}_{\text{ads}}$ is pertained to the equilibrium constant of the adsorption of the inhibitor. It could be computed by the next relation (11) [51]:

12 $${\text{K}}_{{{\text{ads}}}} = {1}/{55}.{\text{5 exp }}( - (\Delta {\text{G}}_{{{\text{ads}}}} /{\text{R}}.{\text{T}}.)$$

where ∆${\text{G}}_{\text{ads}}$ represents the standard free energy of inhibitor adsorption, 55.5 represents the molar concentration of water in the solution, R represents the gas constant, and T represents the absolute temperature. Using the prior equation, the standard Gibbs free energy of the inhibitor’s adsorption at 298 K was calculated and summarized in Table S16. When ∆${\text{G}}_{\text{ads}}$ is approximately − 20 kJ ${\text{mol}}^{-1}$ or below, adsorption is caused by the electrostatic contact between the charged molecules of the inhibitors and the charged electrode (physic-sorption). Those greater than − 40 kJ ${\text{mol}}^{-1}$ demonstrate the charge transfer from the inhibitor (the prepared polymers) to the metal surface (chemisorption) [52]. In this study, the negative values of ∆${\text{G}}_{\text{ads}}$ (less than − 20 kJ ${\text{mol}}^{-1}$) indicate that the adsorption process of the developed inhibitors on CADI alloys in 1M H_2_SO_4_ solution is spontaneous and that the adsorption method of the researched inhibitors follows physic-sorption.

### Temkin adsorption isotherm

As established in Eqs. 13 and 14 [53], the degree of surface covering (*θ*) is related to the inhibitor concentration (C) and the adsorption equilibrium constant *K*_ads_.

13 $${\text{exp}}^{{( - {2}a\theta )}} = K_{{{\text{ads}}}} \times C$$

14 $$\uptheta = - \frac{2.303}{{2{\text{a}}}}\log K - \frac{2.303}{{2{\text{a}}}}\log C$$

where A denotes the attractive parameter, and K is the equilibrium adsorption constant. The linear graphs in Fig. 7b indicate that the adsorption conforms to the Temkin AI. Table S16 shows absorption characteristics derived from this image.

### Freundlich adsorption isotherm (FAI)

Using Eq. 15 [54], the F.A. (*θ)* is linked to the inhibitor concentration C.

15 $${\text{log}}\;\theta = {\text{ log}}\;K_{{{\text{ads}}}} + n{\text{log}}\;C$$

where *n* denotes the empirical constant, the other constants have the same meaning.

Figure 7c depicts a straight-line relationship between log *θ* and log C, with slope n and intercept log *K*_ads_. Table 2 displays the inferred adsorption parameters *K*_ads_, n, and Δ*G*^0^_ads_. The obtained correlation factor values are far from unity. The adsorption process was analyzed using Langmuir, Freundlich, and Temkin isotherms. Adsorption analyses revealed that the experimental findings were linearly compatible with the Langmuir Freundlich and Temkin AIs. The selected criteria for the optimal isotherm are based on the correlation coefficient, R^2^, which is greater. From Table 2 and Fig. 7, LAI with strong correlation coefficients (R) and the Temkin–Freundlich isotherm model provided the best match. K values derived using two-AIs generally agreed with those derived from LAI models.

**Table 2:** Adsorption parameters on CDI alloys as cast with the addition of investigated inhibitors in 1M H_2_SO_4_ solution from W.L. measurements

Isotherm	CADI alloys	R^2^	K_ads_, mol^−1^	∆G_ads_, kJmol^−1^
Langmuir	1% Cr	0.9549	5.0302	− 13.953
	1.5% Cr	0.9604	2.1978	− 11.902
	1% Cr-Nb	0.9930	0.7443	− 9.219
	1.5% Cr-Nb	0.9414	0.2625	− 6.637
Temkin	1% Cr	0.6112	0.6271	− 8.795
	1.5% Cr	0.9934	0.4328	− 7.875
	1% Cr-Nb	0.6076	0.7805	− 9.336
	1.5% Cr-Nb	0.9672	0.1337	− 4.965
Freundlich	1% Cr	0.6043	0.1723	− 5.594
	1.5% Cr	0.9916	0.1163	− 4.620
	1% Cr-Nb	0.5967	0.0818	− 3.748
	1.5% Cr-Nb	0.9752	0.3817	− 7.564

### Corrosion mechanism

Sulfuric acid is a strong acid that dissociates completely in water, increasing the concentration of hydrogen ions in the solution. This high concentration of hydrogen ions accelerates the anodic reaction, leading to rapid metal dissolution. As iron corrodes, it forms ferrous ions, which may further react with oxygen in the presence of moisture to form iron oxides (rust). In sulfuric acid, however, the primary reaction does not typically lead to the formation of iron oxides immediately but rather to the formation of soluble iron ions. Review papers [55] and references have examined and addressed the reactive processes behind the generation of corrosion products. It is possible to evaluate the impact of the formation film or droplets, the creation of corrosion products (dense or compact layers), and the electrolyte flow on corrosion processes and kinetics.

Fe dissolves into Fe^2+^ ions initially as shown in Eq. 5. These ions are subject to hydrolysis (production of Fe(O.H.)^+^) ions) and oxidation (creation of Fe^3+^ ions). Fe(O.H.)^+^ may be rapidly oxidized to Fe(O.H.)_2_^+^, which subsequently becomes Fe(OH)_2_ + (lepidocrocite). This chemical subsequently becomes magnetite (fast oxidation) or green rust (slow oxidation). The production of green rust is followed by the creation of lepidocrocite, which changes into end-products, including goethite (α-FeOOH), magnetite (Fe_3_O_4_), maghemite (γ-Fe_2_O_3_), and hematite (α-Fe_2_O_3_). The final goods’ characteristics rely on environmental circumstances (pH, temperature, solution composition, and oxidation rate). Fe^2+^ based compounds are susceptible to oxidation, whereas Fe^3+^ based compounds are susceptible to reduction (making the oxygen reduction reaction unnecessary and promoting oxidation processes).

### Surface analysis

**Fig. 8:**
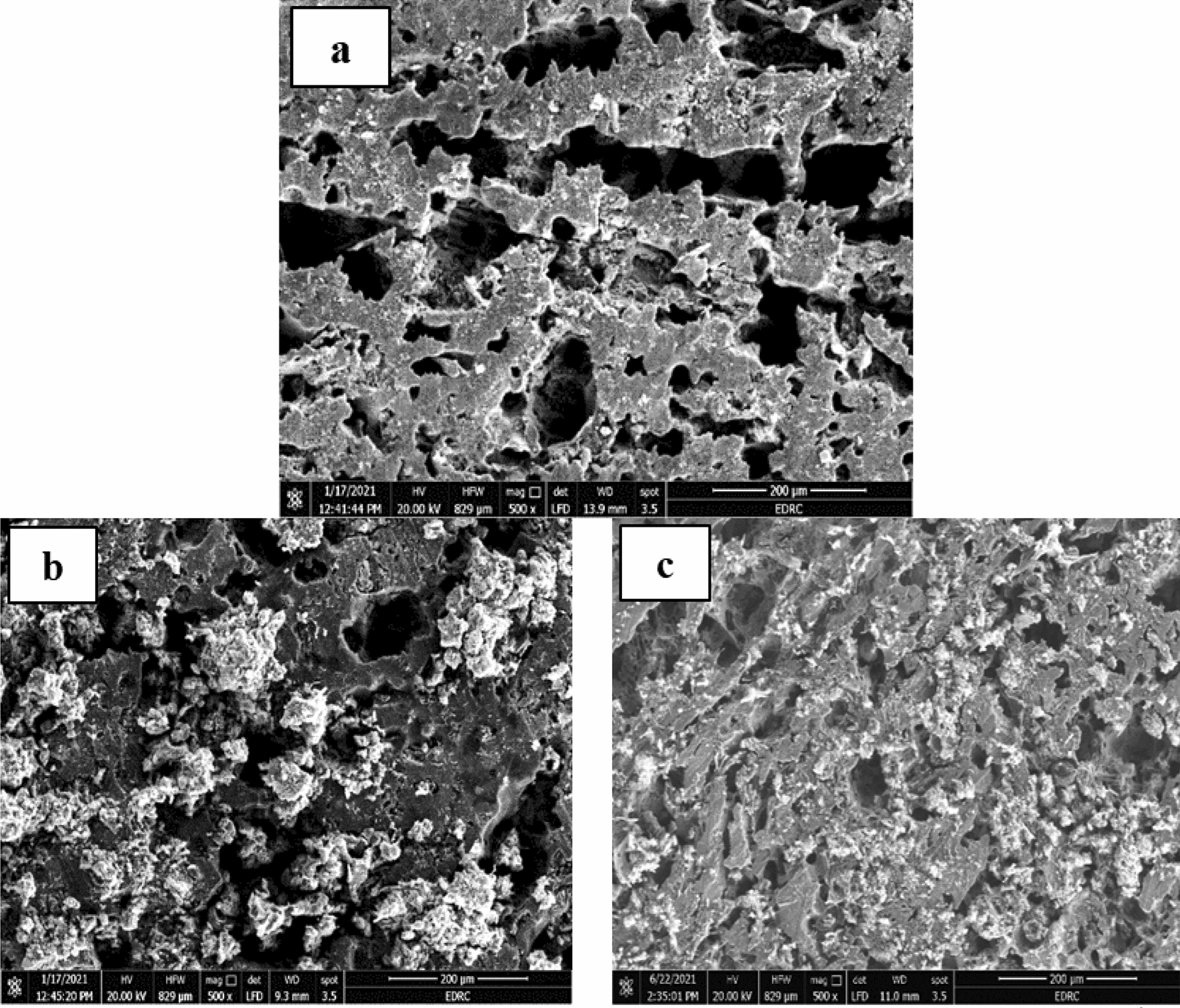
The SEM images of alloys (1.5% Cr) in 1M H_2_SO_4_ **a** As cast, **b** HT-275 °C, and **c** HT-375 °C

**Fig. 9:**
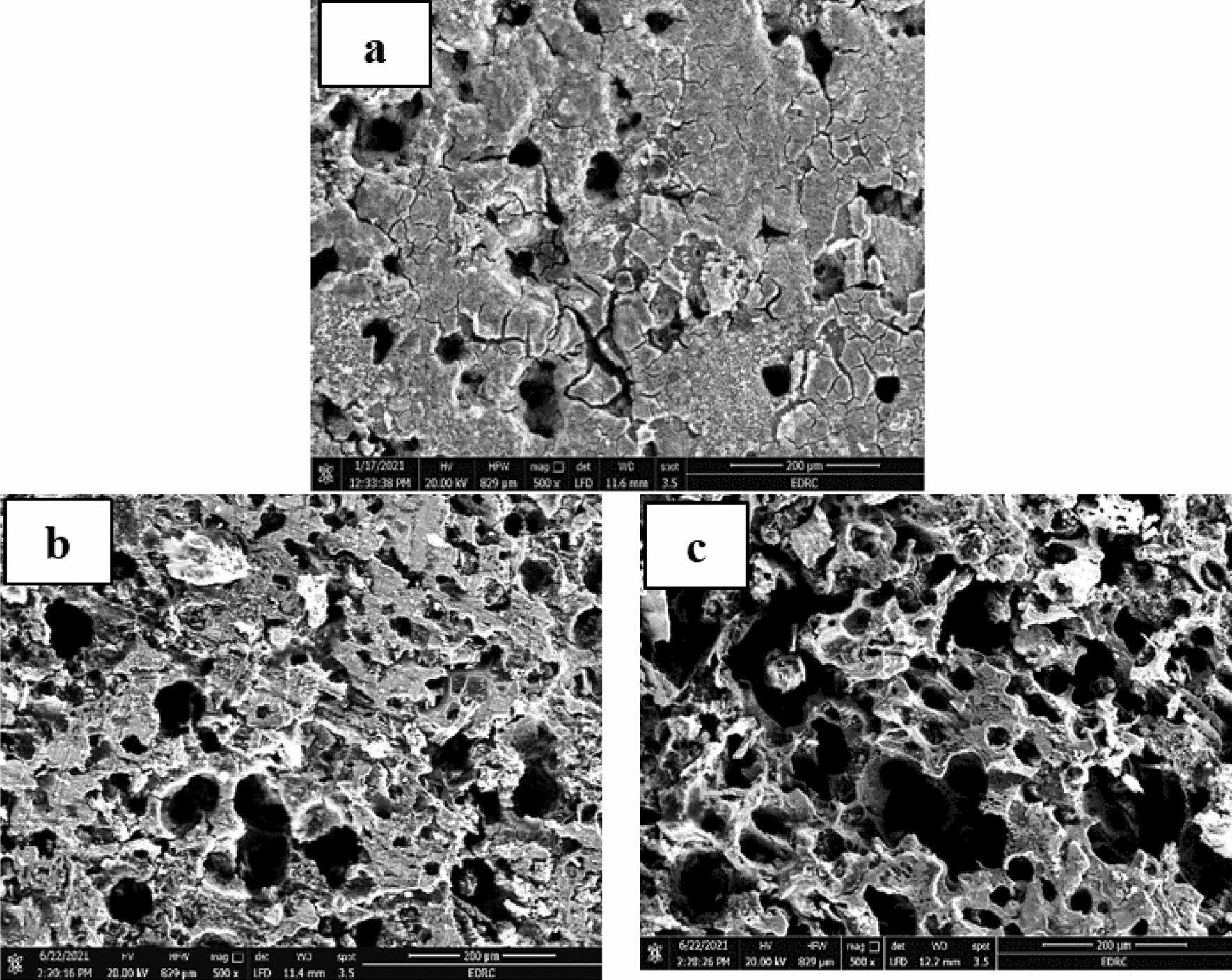
The SEM images of alloys (1.5% Cr–Nb) in 1M H_2_SO_4_ **a** As cast, **b** HT-275 °C, and **c** HT-375 °C

**Fig. 10:**
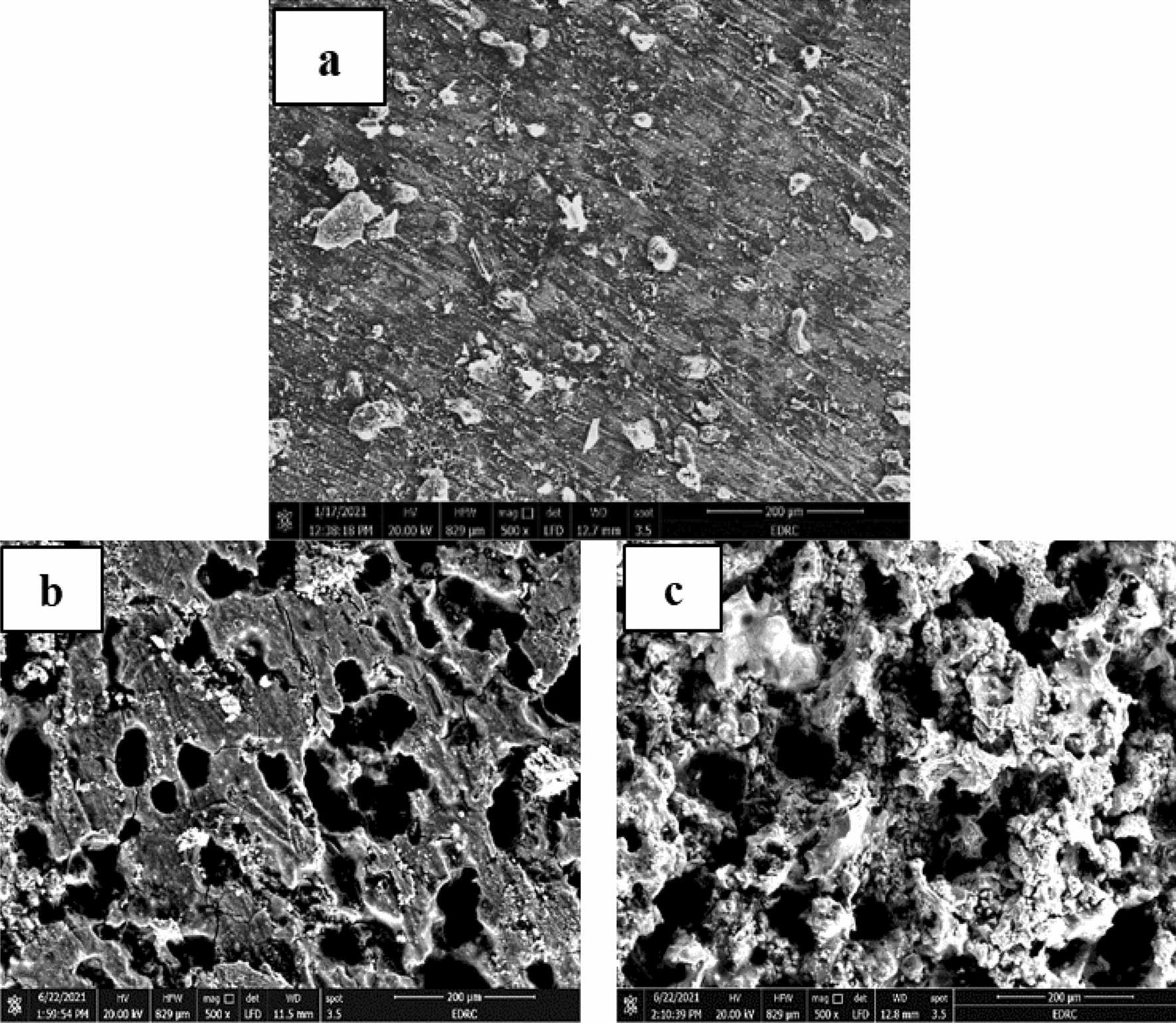
The SEM images of alloys (1.5% Cr –Nb) in 1M H_2_SO_4_ with 100 ppm of investigated inhibitors **a** As cast, **b** HT-275 °C, and **c** HT-375 °C

The surface morphologies of corroded samples show uniform, localized, and galvanic corrosion as shown in Figs. 8, 9, and 10. Uniform corrosion is characterized by a relatively equal breakdown of the metal surface, usually thinning or losing material over the exposed area. Localized corrosion, such as pitting or crevice corrosion, occurs in concentrated parts of the surface that degrade more than the surrounding areas. Galvanic corrosion occurs when two dissimilar metals or alloys electrically connect in a corrosive environment, accelerating corrosion at the interface. The addition of an inhibitor by 100 ppm decreases the CR and enhances the surface morphology. The function of the SEM-studied CADI microstructure and corrosion products in corrosion processes is highlighted. The interaction of inhibitor molecules with the metal surface w, both in the presence and absence of inhibitors, was obtained by SEM morphologies of CADI alloys. These morphologies revealed that the aggressive acid attack severely damaged the CADI alloys immersed in 1 M H_2_SO_4_. After adding the optimal concentration (100 ppm) of inhibitors to a 1M H_2_SO_4_ solution, as seen by SEM images, the surface of the CADI is rather smooth. Based on the SEM pictures, it can be determined that the inhibitor molecules impede the dissolving of steel by creating a layer on the iron's surface. Therefore, the inhibitors defend CADI against the acid solution.

The microstructures of the CADI alloys predominantly consist of ausferrite, which is a microstructural phase comprising acicular ferrite within a stable high-carbon austenite matrix. Additionally, these microstructures contain carbide and nodular graphite, which contribute to the overall properties of the material. Nodular graphite appears scattered throughout the matrix, providing distinct features to the microstructure. Despite the austempering HT being conducted under identical conditions for both CADI 2 alloys (with 1.5% Cr) and CADI 4 alloys (with 1.5% Cr-Nb), noticeable differences are observed in their microstructures when exposed to 1M H_2_SO_4_. Figures 8 and 9 illustrate that both alloy types exhibit an upper ausferritic microstructure characterized by broad ferrite needles. This ausferritic structure, which includes elongated ferrite phases within a high-carbon austenite matrix, significantly impacts the mechanical and corrosion resistance properties of the alloys. The presence of chromium and niobium modifies the structure, resulting in variations in the microstructural appearance and performance under corrosive conditions. The broad ferrite needles observed in the microstructure play a crucial role in influencing the material’s behavior, particularly its resistance to corrosion in sulfuric acid environments.

Due to the inward diffusion of oxygen in the matrix, there is no difference between the graphite spheres and the "ausferrite" matrix. A high density of pits is noted in Fig. 8a. As previously detected in bulk electrolyte solutions [14], pits initiate in the ferrite. Therefore, pitting corrosion and inward oxidation happens during CADI 2 alloys (1.5% Cr). However, the surface is covered with a thick rust layer because of the outward diffusion of metallic ions. Again, there is no significant difference between the rust layer and graphite spheres, as shown in Fig. 9, because of the inward diffusion of oxygen in the matrix. As seen in Fig. 9a, some graphite spheres include small fissures. The dense rust layer can prohibit further CADI corrosion. The evolution of the W.L. measurements proved this.

In contrast, the CADI 4 alloys (1.5% Cr–Nb) in 1M H_2_SO_4_ with 100 ppm of the studied inhibitors have a reduced ausferrite microstructure with small ferrite needles as shown in Fig. 10. Adding Cr, Nb, and inhibitors may efficiently refine grains and promote the production of acicular ferrite [56]. It is known that Nb may exist in acicular ferrite as well as carbide. During austenitization, the austenite’s Nb reduces the carbon diffusion rate, and the discrete distribution of carbides inhibits grain expansion. As the Nb concentration grew, so did the amount of austenite and carbides, while the acicular ferrite got finer and corrosion resistance improved [56]. However, CADI's corrosion resistance may be increased by adding alloying elements and high temperatures [57]. Nb improves the graphite morphology and matrix structure of DI. In addition, Nb may efficiently purify grains and stimulate the production of acicular ferrite; accordingly, improved characteristics are anticipated.

### Theoretical studies of CMBAH inhibitor

**Fig. 11:**
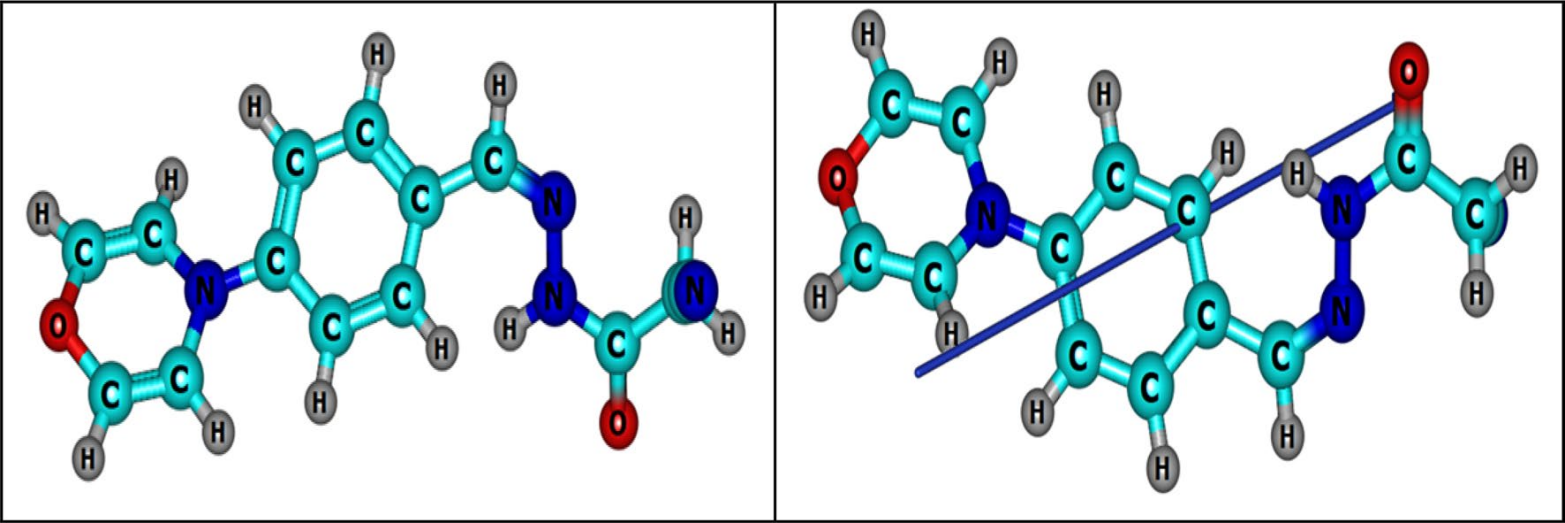
Optimized structure and dipole moment vector of inhibitor (CMBAH)

**Fig. 12:**
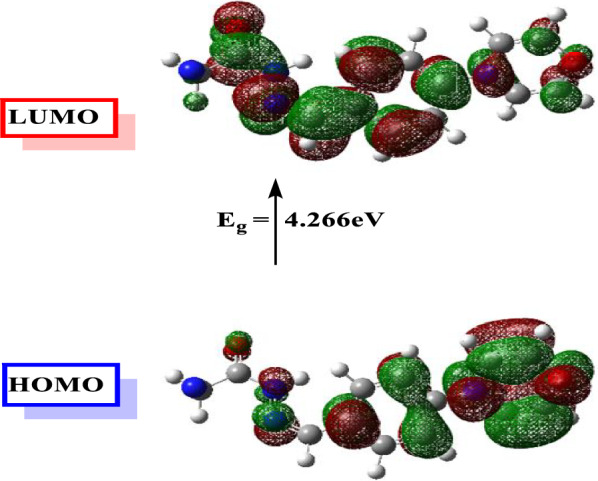
MO and their energies for CMBAH inhibitor

Figures 11 and 12 exhibit the optimized molecular structures and associated maximum occupied frontier molecular orbital (HOMO) and lowest unoccupied frontier molecular orbital (LUMO) for the examined inhibitor. Percent inhibition efficiencies are related to the HOMO and LUMO energies. The % inhibition efficiencies rise if the molecules’ HOMO energies are greater and their LUMO energies are lower [58–60]. The percentage of inhibitory efficiency grew as the energy gap (ΔE) decreased. The EHOMO, ELUMO, and ΔE results indicate that CMBAH has a greater capacity to suppress corrosion. Several other parameters, including global hardness (η), global softness (σ), electronegativity (*X*), chemical potential (u), global electrophilicity (ω), the fraction of electron transfer (ΔN), and energy associated with a backing donation (E_b−d_), are discussed in Table 3 to provide additional insight into the interaction between the inhibitor molecule and the metal surface. Moreover, our works have already given the connections used in these computations [18, 61, 62].

**Table 3:** Quantum chemical parameters calculated for CMBAH inhibitor

Parameters	CMBAH
Total energy, (Hartree)	− 910.001
Dipole moment, (Debye)	6.3123
Chemical potential u, (eV)	− 4.269
electronegativity *X*, (eV)	4.269
E_HOMO_, (eV)	− 6.402
E_LUMO_, (eV)	− 2.136
ΔE, (eV)	4.266
η, (eV)	2.133
σ, (eV^−1^)	0.4688
∆N	0.6401
ω	4.2720
E_b−d_ (eV)	− 0.5332

In general, an inhibitor with a low global hardness and a high softness value has strong chemical reactivity and high inhibitory effectiveness [17, 63]. In addition, as shown in Table 3, the chemical potential values of all S.B. are negative, indicating that they are stable. In addition, the CMBAH inhibitor’s high potential value (− 4.269 eV) and low electrophilicity value (4.2720) encourage its nucleophilic activity [64, 65]. This conclusion follows the HOMO and LUMO energy values [66–68].

The proportion of electron transfer (ΔN) is used to show a molecule’s capacity to absorb or transmit electrons to or from a metal. If ΔN > 0, the inhibitor may give its electron to the metal, and the opposite is true if ΔN < 0 [69]. Consequently, the positive values of ΔN for the investigated inhibitor indicate that electron donation occurs from the inhibitor to the metal surface. Back donation from the metal to the inhibitor is energetically beneficial, as shown by the negative sign of E_b−d._ The back contribution and donation procedures promote inhibitors’ adsorption on the iron surface. These findings are consistent with the experimental effectiveness of inhibition. The electrostatic potential of molecules is utilized to anticipate the reactivity of inhibitor compounds and the overall distribution of charges, as in Fig. S5. In the resulting MEP, the blue (positive) areas describe the love electron sites, while the red (negative) portions describe the love nucleus attack.

## Conclusion

This study explored the inhibitory effectiveness of Schiff base-derived compounds, particularly CMBAH, on the corrosion of CADI alloys in 1M H_2_SO_4_ using both experimental chemical measurements and theoretical DFT calculations. In the acidic environment (1M H_2_SO_4_) over 5 days, CDI alloys demonstrated greater corrosion resistance compared to CADI-HT, attributed to phase transformations achieved through heat treatment. For CDI 4 (1.5% Cr-Nb), the corrosion rate (C.R.) was 11.69 mm/y for the as-cast alloy, decreasing to 5.31 mm/y at HT-275 °C and 6.126 mm/y at HT-375 °C. The Langmuir adsorption isotherm (LAI) effectively describes the inhibitor adsorption, with ∆G_ads_ values indicating spontaneous adsorption and adherence to physisorption. The heat-treated samples mainly consist of nodular graphite, acicular ferrite, austenite, and carbides. Comparing corrosion rates in 1M H_2_SO_4_ with 50 ppm inhibitors for CDI4 and CADI 4 (1.5% Cr-Nb), the rates were 10.22 mm/y as-cast, 4.98 mm/y at HT-275 °C, and 3.66 mm/y at HT-375 °C. CADI alloyed with Nb shows a thicker acicular ferrite, while CADI alloyed with Nb and inhibitors results in a finer acicular ferrite microstructure, which enhances corrosion resistance in harsh 1M H_2_SO_4_ environments. Both theoretical and experimental results align well. The surface morphologies of the different corroded samples provide uniform corrosion, localized corrosion, and galvanic corrosion. Despite the reduction in CR observed with the addition of inhibitors to CADI, there remain limitations and challenges that require further investigation. Future research is needed to explore the long-term stability of these inhibitors, their interaction with various environments, and potential improvements in their efficiency to address the remaining challenges in corrosion protection.

## Supplementary Information

Supplementary Material 1

## Data Availability

The datasets used and/or analysed during the current study are available from the corresponding author on reasonable request.
