# Supplementary material for: Corrosion potential and theoretical studies of fabricated Schiff base for carbidic austempered ductile iron in 1M H2SO4 solution
Source: BMC Chem. 2024 Sep 13;18(1):170. doi: 10.1186/s13065-024-01278-0 (PMC11401432; doi:10.1186/s13065-024-01278-0)
Supplement: Supplementary file 1 — Supplementary Material 1 [file 13065_2024_1278_MOESM1_ESM.docx]

**Corrosion Potential and Theoretical Studies of Fabricated Schiff Base for Carbidic Austempered Ductile Iron in 1M H_2_SO_4_ Solution**

**Ghalia A. Gaber ^1,*^, Lamiaa Z. Mohamed^2^, Hayam. A. Aly^3,4^ , Shimaa Hosny^5, *^**

^1^ Department of Chemistry, Faculty of Science (Girls), Al-Azhar University, P.O. Box: 11754, Yousef Abbas Str., Nasr City, Cairo, Egypt

^2^ Mining, Petroleum, and Metallurgical Engineering Department, Faculty of Engineering, Cairo University, Giza, 12613, Egypt

^3^ Central Metallurgical Research and Development Institute (CMRDI), P.O. Box 87, Helwan, Cairo, 11421, Egypt

^4^ Department of Metallurgical and Materials Engineering, Faculty of Petroleum and Mining Engineering, Suez University, Suez 43512, Egypt

^5^ Chemistry Department, Faculty of Science, New Valley University, El-Kharga, 72511, Egypt

*Corresponding author's E-mail address

[**ghaliaasaid@azhar.edu.eg**](mailto:ghaliaasaid@azhar.edu.eg)**,** [**shimaahosny@sci.nvu.edu.eg**](mailto:shimaahosny@sci.nvu.edu.eg)

**
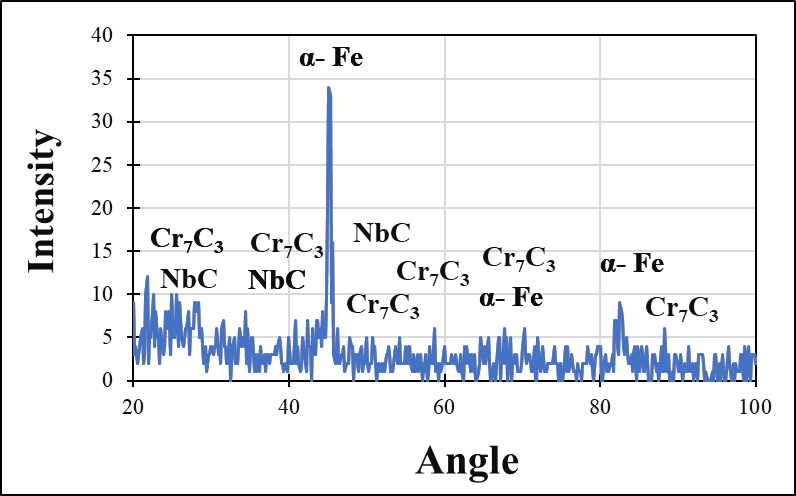
**

**Fig. S1.** XRD pattern of CDI 4 alloy.

**Table S1.** Corrosion parameters obtained from W.L. measurement of CADI alloys in 1M H_2_SO_4_ after 5 days immersion time.

| **CADI alloys** | | | | **Conditions** | |
| --- | --- | --- | --- | --- | --- |
| **1.5 % Cr-Nb** | **1 % Cr-Nb** | **1.5 % Cr** | **1 % Cr** |  |  |
| 0.46 | 0.48 | 0.54 | 0.59 | WL (g) | As cast |
| 11.69 | 12.36 | 13.90 | 14.90 | CR (mm/y) |  |
| 0.21 | 0.49 | 0.59 | 0.74 | WL (g) | At 275°C |
| 5.31 | 12.38 | 14.99 | 18.71 | CR (mm/y) |  |
| 0.24 | 0.33 | 0.49 | 0.89 | WL (g) | At 375°C |
| 6.13 | 8.47 | 12.56 | 22.50 | CR (mm/y) |  |

| 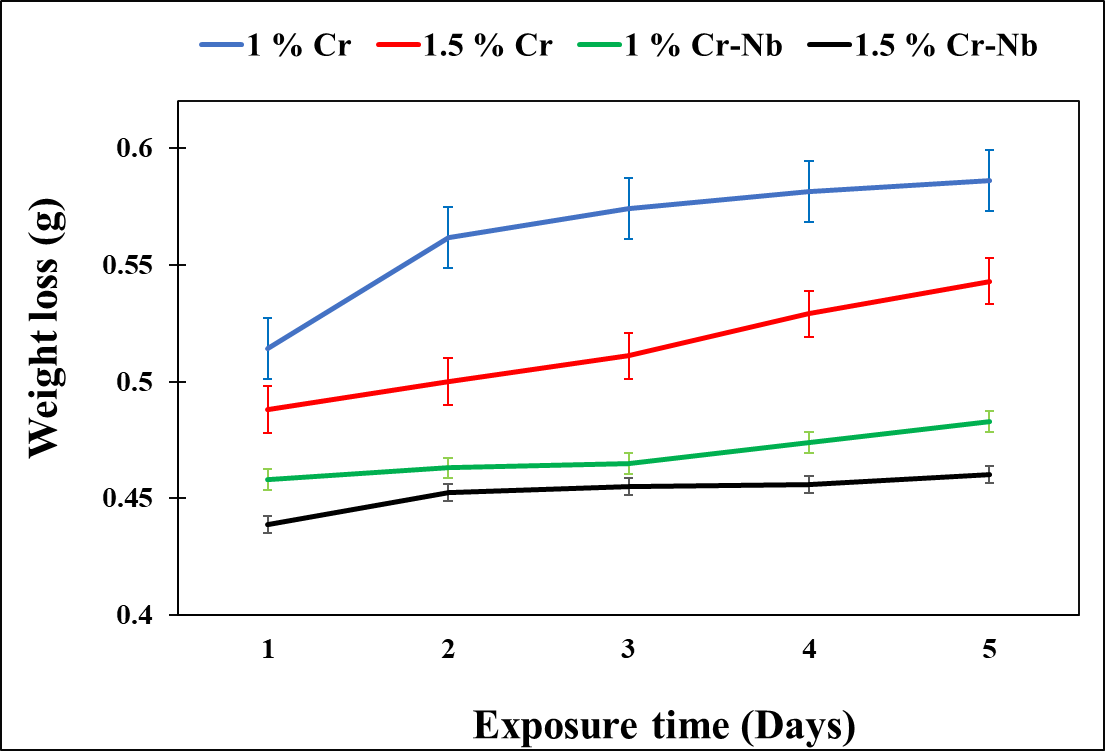 |
| --- |
| (a) |
| 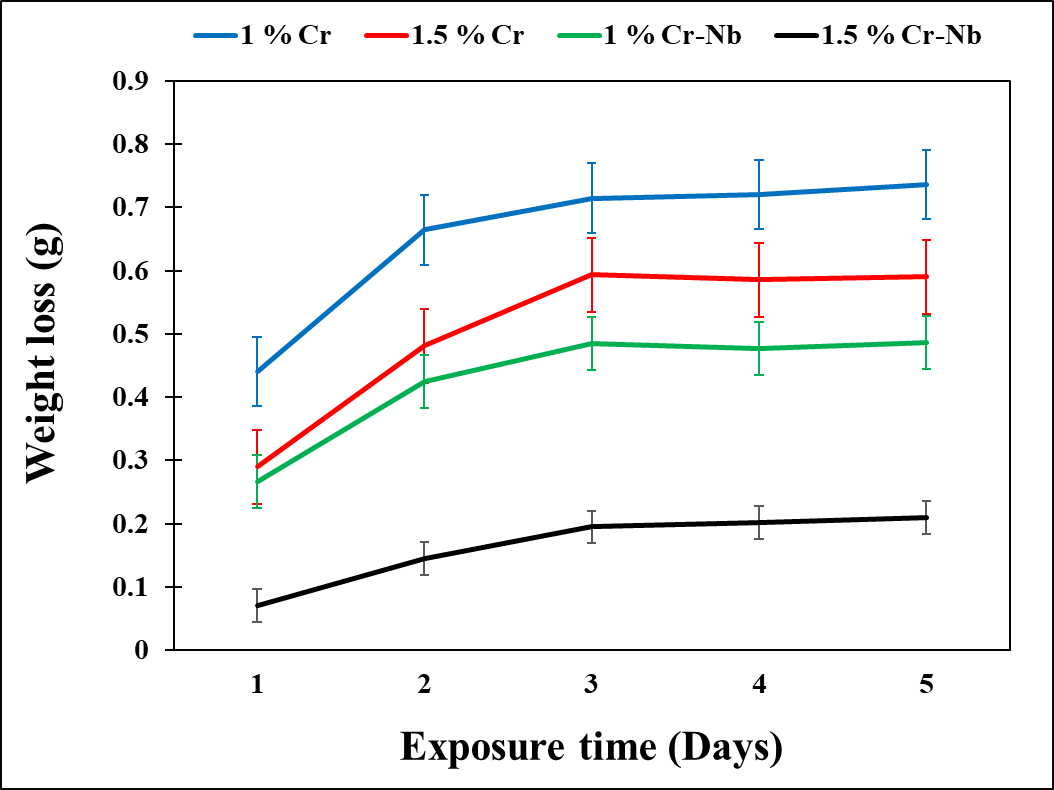 |
| (b) |
| 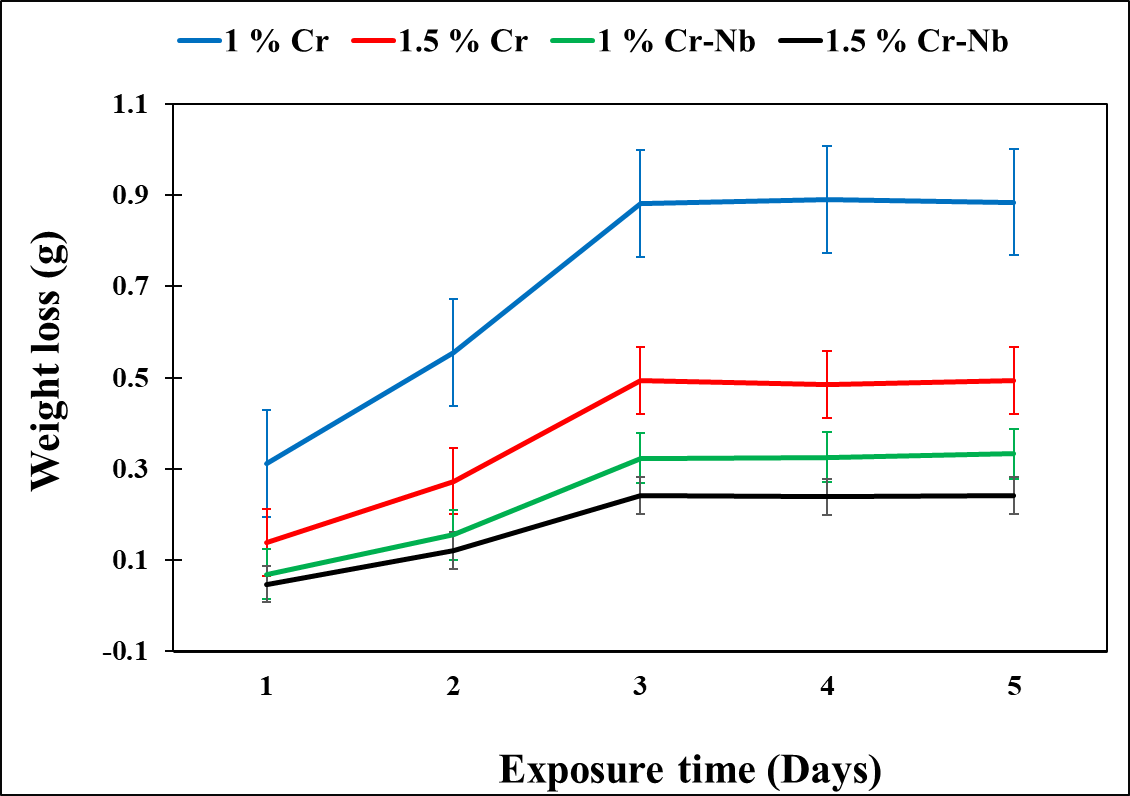 |
| (c) |

**Fig. S2.** WL vs. time for alloys in 1M H_2_SO_4_ (a) As cast, (b) HT-275°C, and (c) HT-375°C.

**Table S2.** W.L. of CDI alloys in 1M H_2_SO_4_ with 50 ppm of inhibitors at different immersion times.

| **Time (Days)** | | | | | **As cast** | |
| --- | --- | --- | --- | --- | --- | --- |
| **5** | **4** | **3** | **2** | **1** |  |  |
| 0.42 | 0.42 | 0.42 | 0.41 | 0.41 | 1 % Cr | CDI 1 |
| 0.41 | 0.41 | 0.41 | 0.41 | 0.41 | 1.5 % Cr | CDI 2 |
| 0.41 | 0.40 | 0.40 | 0.40 | 0.39 | 1 % Cr-Nb | CDI 3 |
| 0.40 | 0.40 | 0.40 | 0.39 | 0.38 | 1.5 % Cr-Nb | CDI 4 |

**Table S3.** W.L. of CADI alloys at HT-275°C in 1M H_2_SO_4_ with 50 ppm of inhibitors at different immersion times.

| **Time (Days)** | | | | | **HT-275°C** | |
| --- | --- | --- | --- | --- | --- | --- |
| 5 | 4 | 3 | 2 | 1 |  |  |
| 0.60 | 0.60 | 0.59 | 0.56 | 0.40 | 1 % Cr | CADI 1 |
| 0.53 | 0.51 | 0.49 | 0.46 | 0.24 | 1.5 % Cr | CADI 2 |
| 0.37 | 0.33 | 0.28 | 0.24 | 0.20 | 1 % Cr-Nb | CADI 3 |
| 0.20 | 0.16 | 0.14 | 0.11 | 0.10 | 1.5 % Cr-Nb | CADI 4 |

**Table S4.** W.L. of CADI alloys at HT-375°C in 1M H_2_SO_4_ with 50 ppm of inhibitors at different immersion times.

| **Time (Days)** | | | | | **HT-375°C** | |
| --- | --- | --- | --- | --- | --- | --- |
| 5 | 4 | 3 | 2 | 1 |  |  |
| 0.69 | 0.69 | 0.58 | 0.35 | 0.26 | 1 % Cr | CADI 1 |
| 0.48 | 0.47 | 0.39 | 0.19 | 0.16 | 1.5 % Cr | CADI 2 |
| 0.29 | 0.28 | 0.19 | 0.12 | 0.10 | 1 % Cr-Nb | CADI 3 |
| 0.14 | 0.13 | 0.13 | 0.09 | 0.05 | 1.5 % Cr-Nb | CADI 4 |

**Table S5.** Corrosion parameters obtained from W.L. measurements of CADI alloys in 1M H_2_SO_4_ with/without 50 ppm of inhibitors after 5 days immersion time.

| **Alloys** | | | | **Conditions** | | |
| --- | --- | --- | --- | --- | --- | --- |
| **CADI 4** | **CADI 3** | **CADI 2** | **CADI 1** |  |  |  |
| 0.46 | 0.48 | 0.54 | 0.59 | WL (g) | 1M H_2_SO_4_ | As  cast |
| 11.69 | 12.36 | 13.90 | 14.90 | CR (mm/y) |  |  |
| 0.40 | 0.41 | 0.41 | 0.42 | WL (g) | 50 ppm |  |
| 10.22 | 10.30 | 10.44 | 10.78 | CR (mm/y) |  |  |
| 0.13 | 0.17 | 0.25 | 0.28 | θ |  |  |
| 12.63 | 16.66 | 24.86 | 27.64 | I.E. % |  |  |
| 0.21 | 0.49 | 0.59 | 0.74 | WL (g) | 1M H_2_SO_4_ | HT-275°C |
| 5.31 | 12.38 | 14.99 | 18.71 | CR (mm/y) |  |  |
| 0.20 | 0.37 | 0.53 | 0.60 | WL (g) | 50 ppm |  |
| 4.98 | 9.41 | 13.37 | 15.35 | CR (mm/y) |  |  |
| 0.06 | 0.24 | 0.1085 | 0.18 | θ |  |  |
| 6.22 | 24.03 | 10.85 | 17.94 | I.E. % |  |  |
| 0.24 | 0.33 | 0.49 | 0.89 | WL (g) | 1M H_2_SO_4_ | HT-375°C |
| 6.13 | 8.47 | 12.56 | 22.50 | CR (mm/y) |  |  |
| 0.14 | 0.29 | 0.4765 | 0.68 | WL (g) | 50 ppm |  |
| 3.66 | 7.42 | 12.1129 | 17.40 | CR (mm/y) |  |  |
| 0.40 | 0.12 | 0.0354 | 0.23 | θ |  |  |
| 40.25 | 12.31 | 3.54 | 22.65 | I.E. % |  |  |

**Table S6.** W.L. of CDI alloys in 1M H_2_SO_4_ with 100 ppm of inhibitors at different immersion times.

| **Time (Days)** | | | | | **As cast** | |
| --- | --- | --- | --- | --- | --- | --- |
| **5** | **4** | **3** | **2** | **1** |  |  |
| 0.42 | 0.42 | 0.41 | 0.41 | 0.40 | 1 % Cr | CDI 1 |
| 0.40 | 0.40 | 0.39 | 0.38 | 0.38 | 1.5 % Cr | CDI 2 |
| 0.38 | 0.37 | 0.36 | 0.36 | 0.35 | 1 % Cr-Nb | CDI 3 |
| 0.36 | 0.35 | 0.34 | 0.33 | 0.33 | 1.5 % Cr-Nb | CDI 4 |

**Table S7.** W.L. of CADI alloys at 275°C in 1M H_2_SO_4_ with 100 ppm of inhibitors at different immersion times.

| **Time (Days)** | | | | | **HT-275°C** | |
| --- | --- | --- | --- | --- | --- | --- |
| **5** | **4** | **3** | **2** | **1** |  |  |
| 0.54 | 0.53 | 0.45 | 0.32 | 0.25 | 1 % Cr | CADI 1 |
| 0.42 | 0.41 | 0.30 | 0.20 | 0.12 | 1.5 % Cr | CADI 2 |
| 0.31 | 0.27 | 0.19 | 0.12 | 0.07 | 1 % Cr-Nb | CADI 3 |
| 0.14 | 0.10 | 0.06 | 0.03 | 0.01 | 1.5 % Cr-Nb | CADI 4 |

**Table S8.** W.L. of CADI alloys at HT-375°C in 1M H_2_SO_4_ with 100 ppm of inhibitors at different immersion times.

| **Time (Days)** | | | | | **HT-375°C** | |
| --- | --- | --- | --- | --- | --- | --- |
| **5** | **4** | **3** | **2** | **1** |  |  |
| 0.33 | 0.32 | 0.32 | 0.29 | 0.22 | 1 % Cr | CADI 1 |
| 0.25 | 0.24 | 0.22 | 0.20 | 0.19 | 1.5 % Cr | CADI 2 |
| 0.18 | 0.17 | 0.16 | 0.14 | 0.14 | 1 % Cr-Nb | CADI 3 |
| 0.12 | 0.11 | 0.10 | 0.10 | 0.08 | 1.5 % Cr-Nb | CADI 4 |

**Table S9.** Corrosion parameters obtained from W.L. measurement of CADI alloys in 1M H_2_SO_4_ with/without 100 ppm of inhibitors after 5 days immersion time.

| **CADI alloys** | | | | **HT** | | |
| --- | --- | --- | --- | --- | --- | --- |
| **CADI 4** | **CADI 3** | **CADI 2** | **CADI 1** |  |  |  |
| 0.46 | 0.48 | 0.54 | 0.59 | WL (g) | 1M H_2_SO_4_ | As  cast |
| 11.69 | 12.36 | 13.90 | 14.90 | CR (mm/y) |  |  |
| 0.36 | 0.38 | 0.40 | 0.42 | WL (g) | 100 ppm |  |
| 9.05 | 9.77 | 10.20 | 10.64 | CR (mm/y) |  |  |
| 0.23 | 0.21 | 0.27 | 0.29 | θ |  |  |
| 22.61 | 20.94 | 26.60 | 28.60 | I.E. % |  |  |
| 0.21 | 0.49 | 0.59 | 0.74 | WL (g) | 1M H_2_SO_4_ | HT-275°C |
| 5.31 | 12.38 | 14.99 | 18.71 | CR (mm/y) |  |  |
| 0.14 | 0.31 | 0.42 | 0.54 | WL (g) | 100 ppm |  |
| 3.55 | 7.95 | 10.74 | 13.79 | CR (mm/y) |  |  |
| 0.33 | 0.36 | 0.28 | 0.26 | θ |  |  |
| 33.11 | 35.81 | 28.42 | 26.29 | I.E. % |  |  |
| 0.24 | 0.33 | 0.49 | 0.89 | WL (g) | 1M H_2_SO_4_ | HT-375°C |
| 6.13 | 8.47 | 12.56 | 22.50 | CR (mm/y) |  |  |
| 0.12 | 0.18 | 0.25 | 0.33 | WL (g) | 100 ppm |  |
| 3.06 | 4.52 | 6.33 | 8.26 | CR (mm/y) |  |  |
| 0.51 | 0.47 | 0.50 | 0.63 | θ |  |  |
| 50.62 | 46.55 | 49.59 | 63.27 | I.E. % |  |  |

| 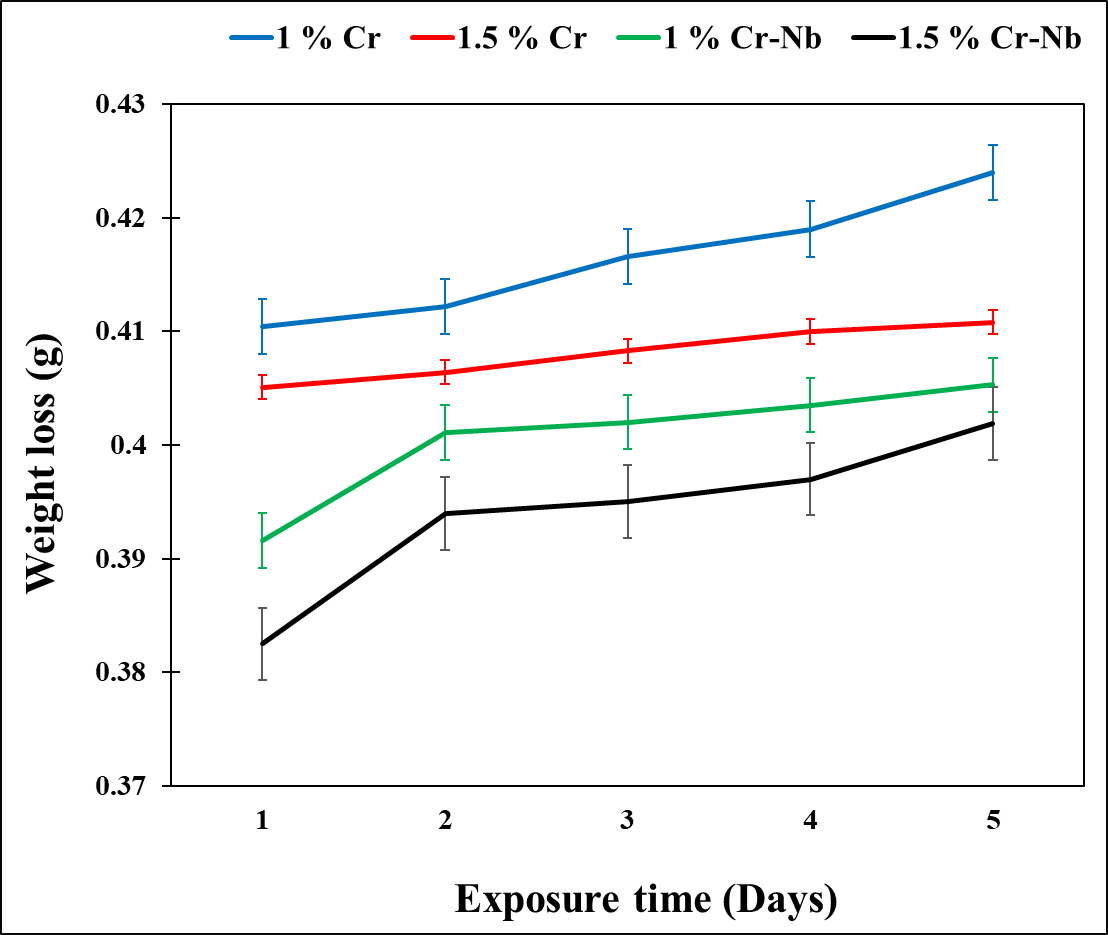 |
| --- |
| (a) |
| 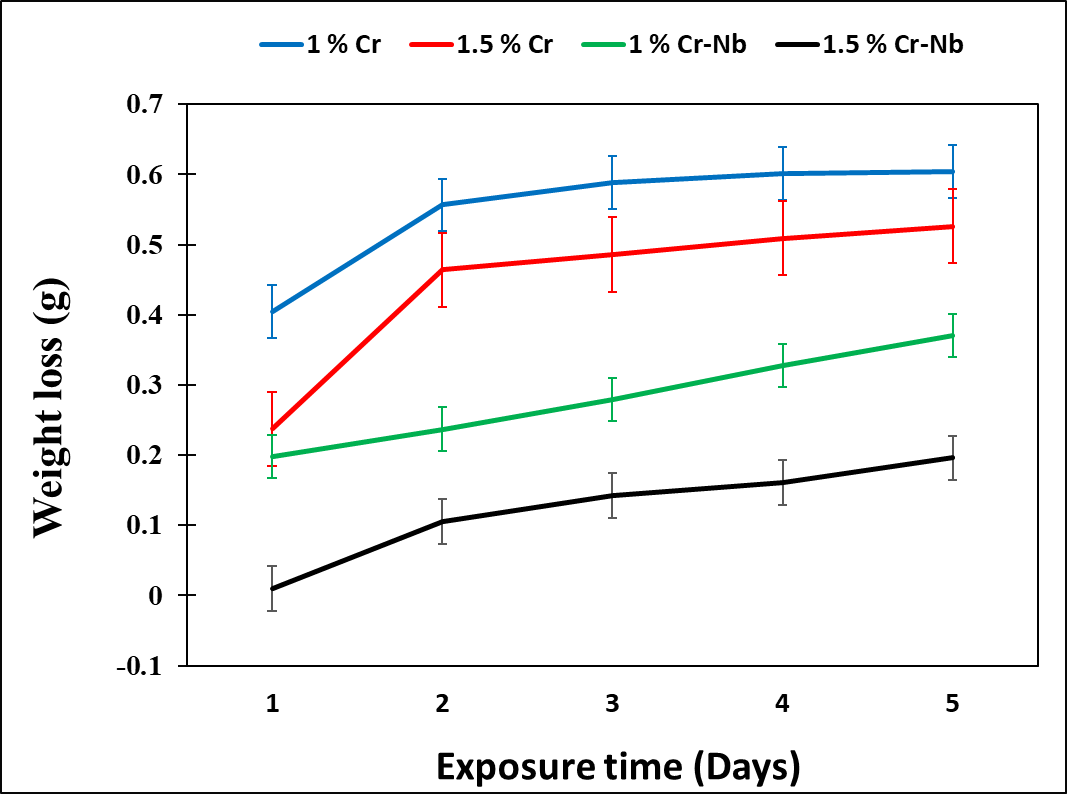 |
| (b) |
| 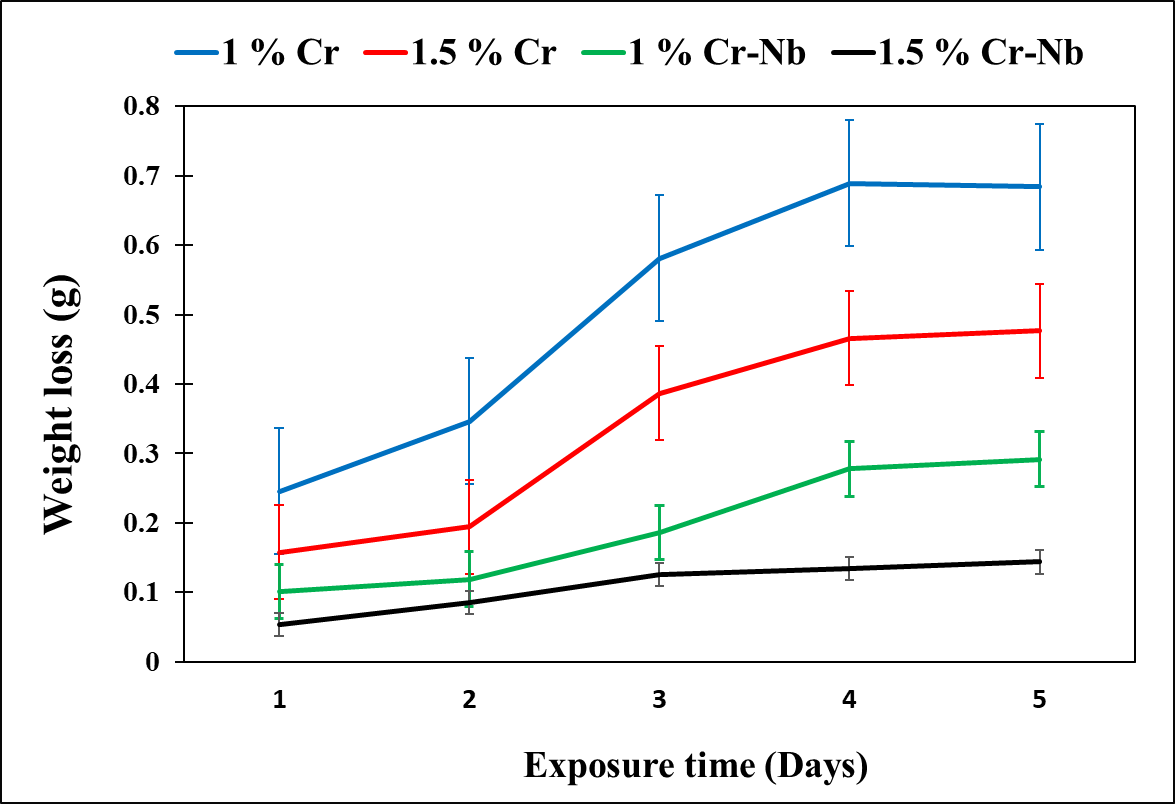 |
| (c) |

**Fig. S3.** W.L. vs. time for alloys in 1M H_2_SO_4_ with 50 ppm of investigated inhibitor (a) As cast, (b) HT-275°C, and (c) HT-375°C.

| 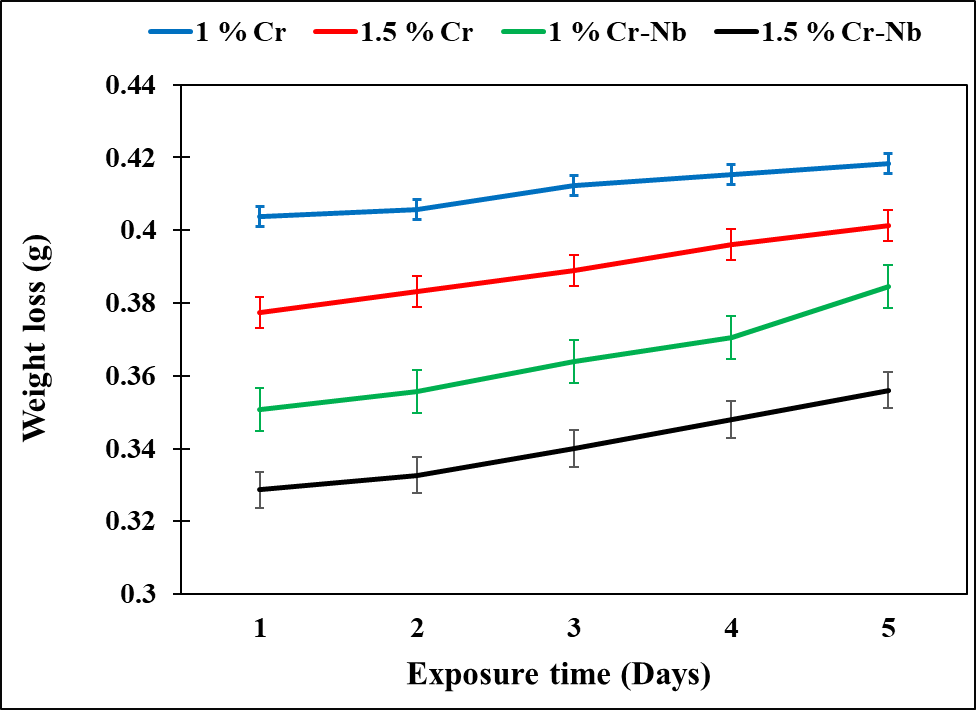 |
| --- |
| (a) |
| 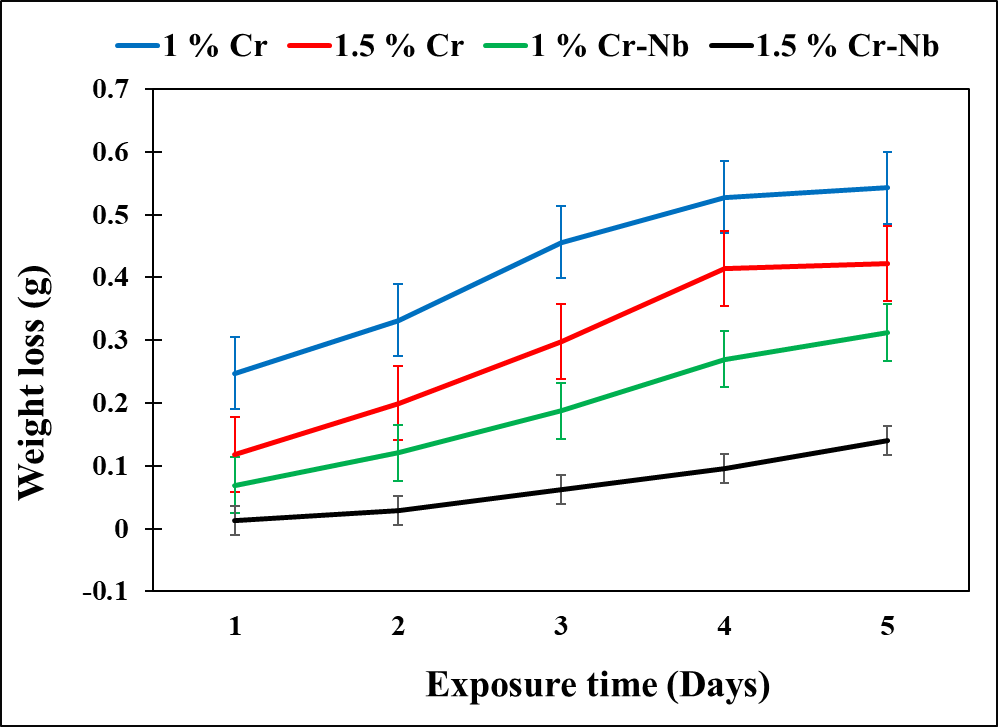 |
| (b) |
| 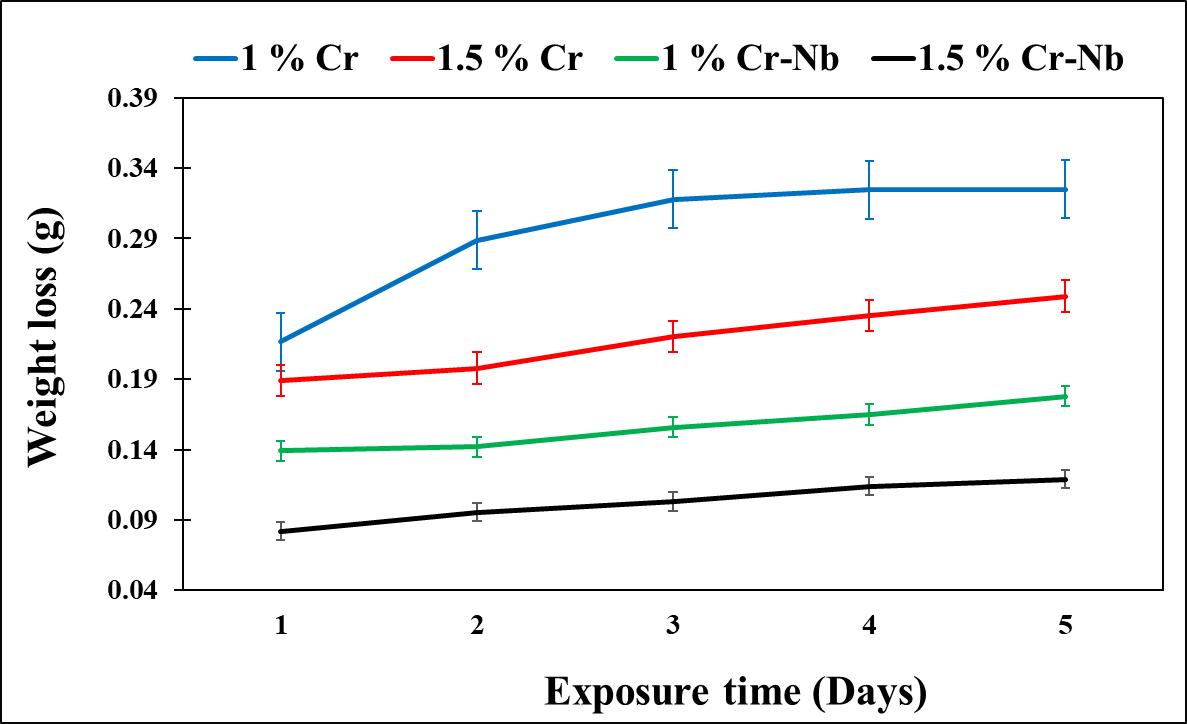 |
| (c) |

**Fig. S4.** W.L. vs. time for alloys in 1M H_2_SO_4_ with 100 ppm of investigated inhibitor (a) As cast, (b) HT-275°C, and (c) HT-375°C.

**Table S10.** Corrosion parameters of CDI alloys in 1M H_2_SO_4_ solution.

| **As cast** | | ***E_corr_***  **mV** | ***I_corr_***  **mA/cm^2^** | ***β_a_***  **mV/dec** | ***β_c_***  **mV/dec** | ***C.R.* mm/y** |
| --- | --- | --- | --- | --- | --- | --- |
| CDI 1 | 1 % Cr | -437.9 | 17.5042 | 253.0 | -196.0 | 204.7 |
| CDI 2 | 1.5 % Cr | -367.4 | 15.0540 | 214.6 | -187.6 | 176.0 |
| CDI 3 | 1 % Cr-Nb | -387.8 | 12.8623 | 197.9 | -196.0 | 150.4 |
| CDI 4 | 1.5 % Cr-Nb | -412.3 | 5.2738 | 97.5 | -152.1 | 61.68 |

**Table S11.** Corrosion parameters of CADI alloys at HT-275°C in 1M H_2_SO_4_ solution.

| **HT-275°C** | | ***E_corr_***  **mV** | ***I_corr_***  **mA/cm^2^** | ***β_a_***  **mV/dec** | ***β_c_***  **mV/dec** | ***C.R.* mm/y** |
| --- | --- | --- | --- | --- | --- | --- |
| CADI 1 | 1 % Cr | -466.0 | 13.4903 | 163.4 | -206.8 | 157.7 |
| CADI 2 | 1.5 % Cr | -428.2 | 11.6816 | 178.6 | -186.3 | 136.6 |
| CADI 3 | 1 % Cr-Nb | -435.2 | 5.7472 | 172.1 | -145.9 | 67.22 |
| CADI 4 | 1.5 % Cr-Nb | -361.0 | 3.3280 | 97.4 | -162.0 | 38.92 |

**Table S12.** Corrosion parameters of CADI alloys at HT-375°C in 1M H_2_SO_4_ solution.

| **HT-375°C** | | ***E_corr_***  **mV** | ***I_corr_***  **mA/cm^2^** | ***β_a_***  **mV/dec** | ***β_c_***  **mV/dec** | ***C.R.* mm/y** |
| --- | --- | --- | --- | --- | --- | --- |
| CADI 1 | 1 % Cr | -469.4 | 16.5758 | 179.9 | -190.0 | 193.8 |
| CADI 2 | 1.5 % Cr | -458.5 | 14.1679 | 237.5 | -187.0 | 165.7 |
| CADI 3 | 1 % Cr-Nb | -436.0 | 7.8818 | 216.7 | -201.0 | 92.18 |
| CADI 4 | 1.5 % Cr-Nb | -444.7 | 2.7237 | 86.0 | -109.8 | 31.85 |

**Table S13.** Corrosion parameters of CDI alloys in 1M H_2_SO_4_ with 100 ppm of inhibitors.

| **As cast** | | ***E_corr_***  **mV** | ***I_corr_***  **mA/cm^2^** | ***β_a_***  **mV/dec** | ***β_c_***  **mV/dec** | ***C.R.* mm/y** |
| --- | --- | --- | --- | --- | --- | --- |
| CDI 1 | 1 % Cr | -428.0 | 8.3265 | 197.9 | -192.0 | 97.38 |
| CDI 2 | 1.5 % Cr | -371.3 | 3.86748 | 203.7 | -199.7 | 45.23 |
| CDI 3 | 1 % Cr-Nb | -383.6 | 2.94945 | 214.4 | -185.4 | 34.49 |
| CDI 4 | 1.5 % Cr-Nb | -413.7 | 2.42258 | 266.4 | -188.2 | 28.33 |

**Table S14.** Corrosion parameters of CADI alloys at 275°C in 1M H_2_SO_4_ with 100 ppm of inhibitors.

| **HT-275°C** | | ***E_corr_***  **mV** | ***I_corr_***  **mA/cm^2^** | ***β_a_***  **mV/dec** | ***β_c_***  **mV/dec** | ***C.R.* mm/y** |
| --- | --- | --- | --- | --- | --- | --- |
| CADI 1 | 1 % Cr | -435.3 | 5.51060 | 79.5 | -165.9 | 64.45 |
| CADI 2 | 1.5 % Cr | -426.4 | 2.47350 | 190.6 | -196.2 | 28.93 |
| CADI 3 | 1 % Cr-Nb | -440.1 | 2.18840 | 184.1 | -193.6 | 25.59 |
| CADI 4 | 1.5 % Cr-Nb | -453.1 | 1.52215 | 179.8 | -201.7 | 17.80 |

**Table S15.** Corrosion parameters of CADI alloys at HT-375°C in 1M H_2_SO_4_ with 100 ppm of inhibitors.

| **HT-375°C** | | ***E_corr_***  **mV** | ***I_corr_***  **mA/cm^2^** | ***β_a_***  **mV/dec** | ***β_c_***  **mV/dec** | ***C.R.* mm/y** |
| --- | --- | --- | --- | --- | --- | --- |
| CADI 1 | 1 % Cr | -445.2 | 6.87250 | 83.4 | -169.4 | 80.38 |
| CADI 2 | 1.5 % Cr | -452.5 | 2.18110 | 92.9 | -186.5 | 25.51 |
| CADI 3 | 1 % Cr-Nb | -425.6 | 1.82668 | 120.0 | -178.4 | 21.36 |
| CADI 4 | 1.5 % Cr-Nb | -418.5 | 1.60531 | 197.2 | -199.5 | 18.77 |

**Table S16.** Inhibition efficiency and surface coverage obtained from the PDP method of CADI alloys in 1M H_2_SO_4_ with/without 100 ppm of inhibitors.

| **CADI alloys** | | | | **HT** | | |
| --- | --- | --- | --- | --- | --- | --- |
| **CADI 4** | **CADI 3** | **CADI 2** | **CADI 1** |  |  |  |
| 61.68 | 150.4 | 176.0 | 204.7 | CR (mm/y) | 1M H_2_SO_4_ | As  cast |
| 28.33 | 34.49 | 45.23 | 97.38 | CR (mm/y) | 100 ppm |  |
| 0.541 | 0.771 | 0.743 | 0.524 | θ |  |  |
| 54.1 | 77.1 | 74.3 | 52.4 | IE % |  |  |
| 38.92 | 67.22 | 136.6 | 157.7 | CR (mm/y) | 1M H_2_SO_4_ | HT-275°C |
| 17.80 | 25.59 | 28.93 | 64.45 | CR (mm/y) | 100 ppm |  |
| 0.543 | 0.619 | 0.788 | 0.591 | θ |  |  |
| 54.3 | 61.9 | 78.8 | 59.1 | IE % |  |  |
| 31.85 | 92.18 | 165.7 | 193.8 | CR (mm/y) | 1M H_2_SO_4_ | HT-375°C |
| 18.77 | 21.36 | 25.51 | 80.38 | CR (mm/y) | 100 ppm |  |
| 0.411 | 0.768 | 0.846 | 0.585 | θ |  |  |
| 41.1 | 76.8 | 84.6 | 58.5 | IE % |  |  |


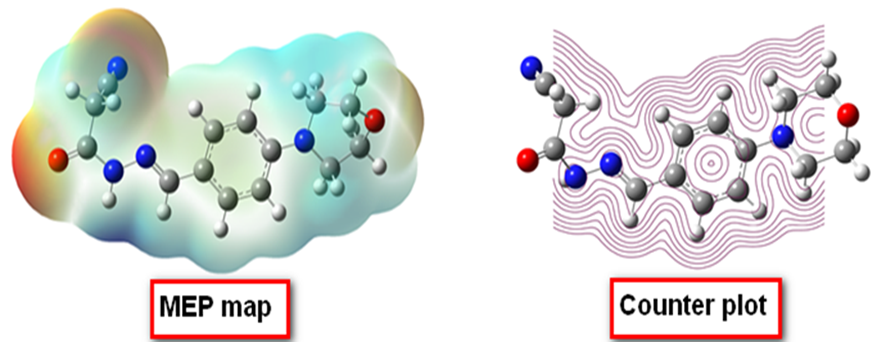


**Fig. S5.** Molecular electrostatic potential (MEP) surface and counterplots for CMBAH inhibitor.
